# Estimating the opportunity costs of bed‐days

**DOI:** 10.1002/hec.3613

**Published:** 2017-11-06

**Authors:** Frank G. Sandmann, Julie V. Robotham, Sarah R. Deeny, W. John Edmunds, Mark Jit

**Affiliations:** ^1^ London School of Hygiene and Tropical Medicine Department of Infectious Disease Epidemiology London UK; ^2^ Public Health England Modelling and Economics Unit London UK; ^3^ The Health Foundation London UK

**Keywords:** economic evaluation, healthcare costs, length of stay, net benefit, opportunity costs

## Abstract

Opportunity costs of bed‐days are fundamental to understanding the value of healthcare systems. They greatly influence burden of disease estimations and economic evaluations involving stays in healthcare facilities. However, different estimation techniques employ assumptions that differ crucially in whether to consider the value of the second‐best alternative use forgone, of any available alternative use, or the value of the actually chosen alternative. Informed by economic theory, this paper provides a taxonomic framework of methodologies for estimating the opportunity costs of resources. This taxonomy is then applied to bed‐days by classifying existing approaches accordingly. We highlight differences in valuation between approaches and the perspective adopted, and we use our framework to appraise the assumptions and biases underlying the standard approaches that have been widely adopted mostly unquestioned in the past, such as the conventional use of reference costs and administrative accounting data. Drawing on these findings, we present a novel approach for estimating the opportunity costs of bed‐days in terms of health forgone for the second‐best patient, but expressed monetarily. This alternative approach effectively re‐connects to the concept of choice and explicitly considers net benefits. It is broadly applicable across settings and for other resources besides bed‐days.

## 1 INTRODUCTION

In healthcare settings, resources such as personnel and beds are scarce. Hence, choosing to treat or care for one patient means a lost opportunity to treat or care for another patient in the presence of an unmet demand, as exemplified by waiting lists. It is such a trade‐off that loosely embodies the economic notion of “opportunity costs”.

Most economic textbooks define opportunity costs similar to “the value of the next‐best alternative” forgone, and others consider “(the value of) what is given up” (Ferraro & Taylor, 2005, p.10; Polley, 2015, p.7). Although comparable, the second phrase lacks an explicit valuation ranking (O'Donnell, 2010), that is, it is not clear whether to take the value of the second‐best alternative use, of any available alternative use, or of the actually chosen use. Moreover, the understanding of “costs” also differs between disciplines, with economists focusing on choosing between different possible courses of action given limited resources, and accountants focusing on recovering historical expenditures for financial planning and reporting (Mogyorosy & Smith, 2005). For economists, only factors relating to a sacrifice from making a particular decision are relevant “pain costs” (i.e., costs “felt” by the decision maker), but factors not associated with a sacrifice from that decision are irrelevant “sunk costs” (Buchanan, 1969). This is most obvious for factors fixed in time due to contractual obligations, which cannot be significantly reduced in the short‐term, with variable cost factors becoming the sacrifice of a choice to be made.

Major health economic textbooks endorse valuing opportunity costs with the second‐best alternative forgone (Drummond, Sculpher, Torrance, O'Brien, & Stoddart, 2005; Folland, Goodman, & Stano, 2010; Gray, Clarke, Wolstenholme, & Wordsworth, 2010), particularly for resources without a market price. A “shadow price” is then derived to represent the “true social value (or opportunity cost) of non‐marketed resources, such as time and informal care” (Palmer & Raftery, 1999, p.1552). For marketable resources, however, the market price, reference costs and average accounting expenditure of the *chosen* alternative are conventionally considered to approximate the opportunity costs for pragmatic reasons (Drummond et al., 2005). Explicit consideration of the second‐best alternative is hence dropped and although common practice, this is only adequate under the idealistic market conditions of perfect competition (Buchanan, 1969; Jit et al., 2011).

Given the well‐known market failures in healthcare (Arrow, 1963; Pauly, 1968), this paper scrutinises the estimation techniques that have been adopted in the past mostly without questioning the underlying assumptions and biases. We consider healthcare beds as prime example of an imperfectly‐marketed resource whose opportunity costs may in fact diverge from the values calculated using conventional methods, not least because hospitals are multiproduct firms with a complex production function in which different units may operate internally as individual profit centres. Bed‐days are also a highly influential cost component of any analysis involving stays in healthcare facilities (Drummond et al., 2005). Therefore, we investigated how to adequately estimate the economic value of bed‐days, with a special focus on decision‐making agents aiming to maximise health.

The paper is structured as follows: Our methods and sources are outlined next. In the results, we compile a general taxonomy of methodologies for estimating the opportunity costs of resources. We then focus on the resource “bed‐day” and present co‐existing approaches that we classify according to the taxonomy. To distinguish the theoretical methodologies for resources from the practical applications for bed‐days, we use the terms “methodology” and “approach”, respectively. We illustrate and appraise the approaches before proposing a novel alternative. The proposal and paper are then discussed before offering concluding remarks.

## 2 METHODS

First, theoretical methodologies of estimating opportunity costs were categorised in a taxonomy based on (reviews of) economic textbooks (Drummond et al., 2005; Ferraro & Taylor, 2005; O'Donnell, 2010; Polley, 2014, 2015; Stone, 2015) as well as James Buchanan's treatise on the concept's origins up to the late 1960s and diverging views among orthodox (neo‐classical) and heterodox (subjectivist) schools of economics (Buchanan, 1969).

Second, existing applications for the valuation of bed‐days were identified through a scoping review of the health and economics literature. Relevant articles were initially searched for up to 28 November 2014 in two bibliographic databases: PubMed (NLM) and EconLit (Ovid). Additionally, the reference lists of all articles screened in full‐text were subsequently checked for relevance. The search was last updated on 02 December 2016, using the following search terms: “bed‐day” AND (costs OR demand* OR valu*); “opportunity costs” AND (bed‐day OR health* OR hospital*) for details of the syntax see Table A1 in the Appendix. All articles were included that directly applied an approach to estimate the opportunity costs of bed‐days or one suitable for bed‐days. Records were excluded that did not entail any approach of estimation or any suitable approach for bed‐days, were not written in English, or did not include a full‐text article. Articles using wage rates were also excluded although their relevance to our work is discussed later, and the general idea of multiplying time with a monetary value is incorporated in one of the approaches. The identified applications in the articles were then generalised and clustered into different approaches, which in turn were classified according to our taxonomic framework.

Third, we compared and appraised the different approaches to explore their impact on bed‐day values. Drawing on these findings, we developed a novel approach for valuing the opportunity costs of bed‐days in line with economic theory and from the perspective of a decision maker aiming to maximise health with limited resources.

## 3 RESULTS

### 3.1 General taxonomy of methodologies to estimate the opportunity costs of resources

Early economic theorists initially interpreted opportunity costs in terms of units of a displaced alternative product: “If among a nation of hunters […] it usually costs twice the labour [time] to kill a beaver which it costs to kill a deer, one beaver should naturally exchange for or be worth two deer” (Smith, 1776). In this simplified example, hunting deer is the second‐best alternative for the hunters, and the relative costs of production reflect the true opportunity costs of the hunters' labour time (Buchanan, 1969).

Departing from natural units was seen as necessary by economists to account for the monetary value used in almost all kinds of exchange (Buchanan, 1969; Robbins, 1932). Opportunity costs should then be represented by the net benefit (i.e., benefit minus expenditure; also called the natural or accounting profit) to account for different benefits and expenditures associated with alternative options (O'Donnell, 2010; Polley, 2014). The second‐highest net benefit, that is, the second‐best alternative to choose, constitutes the true opportunity costs (Ferraro & Taylor, 2005; Polley, 2015), with all other alternatives comprising trade‐off costs (O'Donnell, 2010). Moreover, the opportunity costs should not be confused with the economic profit, which is the difference of the highest and second‐highest net benefits (O'Donnell, 2010).

For reasons of practicality, orthodox neoclassical economists then moved to an interpretation of the value of the displaced product being approximated by the expenditure on the alternative chosen. Its costs of production are assumed to reflect the value of a forgone alternative that could have been produced had the same amount of money been spent on it instead (Buchanan, 1969; Drummond et al., 2005). This interpretation is valid for perfectly competitive markets as there will be no alternative, more profitable use of resources at the equilibrium price of supply and demand (i.e., it is “Pareto efficient”; see the first optimality theorem of welfare economics; Folland et al., 2010). It is thus a special situation where the values of all alternatives converge, making net benefits irrelevant in the absence of profitable alternatives as their benefits equal their expenditures.

Due to externalities and information asymmetries, most markets fail to reach the competitive equilibrium (Greenwald & Stiglitz, 1986), including healthcare (Arrow, 1963). Expenditures will then not readily reflect opportunity costs as profits/losses indicate a better use of resources existing elsewhere. Thus in case the optimal alternative is not chosen, the opportunity costs will need to include the optimal profit forgone (Buchanan, 1969). A recent proposal generalised this as adding the incurred expenditure (the “explicit cost”) and the highest net benefit forgone (the “implicit cost”) as opportunity costs (Stone, 2015), which is broadly considered equivalent to “economic costs” (O'Donnell, 2010; Stone, 2015). Yet, this fails to adequately correct for any competitive disequilibrium of the optimal alternative.

Altogether, four different methodologies have evolved over time and comprise our taxonomy:

- Opportunity costs in terms of units of the second‐best alternative forgone;
- Opportunity costs as the net benefit of the second‐best alternative forgone;
- Opportunity costs as the expenditure of the alternative chosen; and
- Opportunity costs as the expenditure of the alternative chosen plus the net benefit of the alternative forgone with the greatest value.

Illustrated with two options *i* and *j*, where *j* is the next‐best alternative to *i*, the value of the marginal opportunity costs of option *i*, OC_*i*_, can be formulated. Marginality (Folland et al., 2010) refers here to the change in costs and benefits (or units of option *j*) when providing/treating one more unit of option *i*. Also, “next‐best” here means “second‐best” when referring to Methodologies A and B, but it means the alternative with the greatest value when referring to Methodology D. The four methodologies then read:

(1) $${\mathrm{OC}}_{i}=u_{j}$$

(2) $${\mathrm{OC}}_{i}={u_{j}}^{*}\left(B_{j}-C_{j}\right)$$

(3) $${\mathrm{OC}}_{i}=C_{i}$$

(4) $${\mathrm{OC}}_{i}=C_{i}+{u_{j}}^{*}\left(B_{j}-C_{j}\right)$$

where *u_j_* denotes the number of units of the next‐best alternative forgone, the net benefit is calculated by subtracting the marginal expenditure of the next‐best alternative, *C*_*j*,_ from its marginal gross benefit, *B_j_*, and *C_i_* is the marginal expenditure of the alternative chosen. Generally, expenditures are a sacrifice, typically of money, and benefits a gain, typically valued as the minimum willingness to trade‐off, that is, pay or sell depending on the perspective, also known as the marginal rate of substitution (Polley, 2015; Stone, 2015), or expressed in health outcomes such as the quality‐adjusted life year, QALY.

As can be seen, Equation (2) extends Equation (1) with the net benefit of the number of units of the second‐best option *j* forgone, and Equation (4) is the sum of Equations (3) and (2).

### 3.2 Existing approaches to estimate the opportunity costs of bed‐days

The scoping review of existing applications suitable to estimate the opportunity costs of bed‐days identified 2,273 records. After the screening and review procedure, 101 relevant articles remained; see Figure A1 in the Appendix. We generalised applications and clustered them into nine approaches, each of which could be classified under one of the four methodologies of our taxonomy. Sixteen articles applied multiple approaches; for a complete list of included references, see Table A2 in the Appendix.

An overview of the nine existing approaches applied for an option (read: patient) *i* is shown in Table 1, together with the results of a numerical illustration and our new proposals presented later. Note that all approaches require information on an alternative option except for those following Methodology C.

**Table 1:** Overview of approaches to value the opportunity costs of bed‐days used for patient *i*

Approach	Description	Equation	Results for patient *i*
Methodology A: Units of the second‐best alternative forgone			
1	Patient‐equivalents (of second‐best patients *j*) forgone	${{\mathrm{LOS}}_{i}}^{*}\frac{1}{{\mathrm{LOS}}_{j}}\left({}^{*}\mathrm{OCR}\right)$	2
2	Treatment‐equivalents forgone for the second‐best patients *j*	${C_{i}}^{*}\frac{1}{C_{j}}$	1.4
Methodology B: Net benefit of the second‐best alternative forgone			
	Valuation in terms of money		
3a	Expenditure forgone on the second‐best patient‐equivalents	${{\mathrm{LOS}}_{i}}^{*}\frac{C_{j}}{{\mathrm{LOS}}_{j}}$	£10,000
3b	Revenue forgone from the second‐best patient‐equivalents	${{\mathrm{LOS}}_{i}}^{*}\frac{R_{j}}{{\mathrm{LOS}}_{j}}\left({}^{*}\mathrm{OCR}\right)$	£12,000
3c	Net revenue forgone from the second‐best patient‐equivalents	${{\mathrm{LOS}}_{i}}^{*}\frac{\left(R_{j}-C_{j}\right)}{{\mathrm{LOS}}_{j}}$	£2,000
5	Gross monetary benefit forgone for the second‐best patient‐equivalents	${{\mathrm{LOS}}_{i}}^{*}\frac{\left({B_{j}}^{*}\lambda \right)}{{\mathrm{LOS}}_{j}}$	£24,000
New1	Net monetary benefit forgone for the second‐best patient‐equivalents	${{\mathrm{LOS}}_{i}}^{*}\frac{\left({B_{j}}^{*}\lambda -C_{j}\right)}{{\mathrm{LOS}}_{j}}$	£14,000
New2	Net monetary benefit forgone for the second‐best treatment‐equivalents	${C_{i}}^{*}\frac{\left({B_{j}}^{*}\lambda -C_{j}\right)}{C_{j}}$	£9,800
	Valuation in terms of health benefit (typically QALYs)		
4	Gross health benefit forgone for second‐best patient‐equivalents	${{\mathrm{LOS}}_{i}}^{*}\frac{B_{j}}{{\mathrm{LOS}}_{j}}$	1.2
6	Health benefit forgone for expected second‐best use	${C_{i}}^{*}\frac{1}{\lambda }$	0.35
New3	Net health benefit forgone for the second‐best patient‐equivalents	${{\mathrm{LOS}}_{i}}^{*}\frac{\left(B_{j}-\left(\frac{C_{j}}{\lambda }\right)\right)}{{\mathrm{LOS}}_{j}}$	0.7
New4	Net health benefit forgone for the second‐best treatment‐equivalents	${C_{i}}^{*}\frac{\left(B_{j}-\left(\frac{C_{j}}{\lambda }\right)\right)}{C_{j}}$	0.49
Methodology C: Expenditure of the alternative chosen			
7	Expenditure for the resource consumption incurred	${{\mathrm{LOS}}_{i}}^{*}\frac{C_{i}}{{\mathrm{LOS}}_{i}}$	£7,000
8	Separating variable expenditure and non‐monetary resource consumption	${{\mathrm{LOS}}_{i}}^{*}\frac{{\mathrm{VC}}_{i}}{{\mathrm{LOS}}_{i}}\&{\mathrm{LOS}}_{i}$	£3,500 & 10
Methodology D: Expenditure of the alternative chosen + highest net benefit forgone			
9	Expenditure incurred + highest net revenue forgone	${{\mathrm{LOS}}_{i}}^{*}\left(\frac{C_{i}}{{\mathrm{LOS}}_{i}}+\frac{\left(R_{j}-C_{j}\right)}{{\mathrm{LOS}}_{j}}\right)$	£9,000
New5	Expenditure incurred + highest net monetary benefit forgone	${{\mathrm{LOS}}_{i}}^{*}\left(\frac{C_{i}}{{\mathrm{LOS}}_{i}}+\frac{\left({B_{j}}^{*}\lambda -C_{j}\right)}{{\mathrm{LOS}}_{j}}\right)$	£21,000

The last column illustrates the marginal opportunity costs of patient *i* consuming 10 bed‐days based on the input data in Table 2. Not all articles used LOS. The equations were rearranged to show how the resource consumption of patient *i* should be valued; we thus included *LOS_i_* in approach 7 and 8 to make this valuation clearer. Our new proposals are labelled with “New”; we favour “New1” (and “New5”, depending on whether the chosen alternative was the sub‐optimal choice) given the minor impact of monetary inflation compared to treatment‐equivalents.

B_j_ = (health) benefit gained per second‐best patient, C_i_ = total expenditure incurred for _i_, C_j_ = expenditure incurred per second‐best patient, λ = monetary value assigned to QALYs in local cost‐effectiveness thresholds, LOS_i_ = total bed‐day consumption of _i_, LOS_j_ = length of stay per second‐best patient, OCR = occupancy rate, QALY = quality‐adjusted life year, R = revenue per patient, VC = variable cost proportion of the expenditure.

#### 3.2.1 Methodology A: Opportunity costs in terms of units of the second‐best alternative forgone

Two approaches used the first methodology by expressing units of the second‐best alternative as patient‐equivalents (Approach 1) or treatment‐equivalents (Approach 2).

Patient‐equivalents were calculated in terms of the number of alternative patients that could have been treated using the same resources, for example, bed‐days, differently. Frequently, the alternative patient was (implicitly) approximated by the average patient population likely to occupy that bed. One article additionally adjusted for an occupancy rate of 0.75 (Coughlan & O'Neill, 2001).

Treatment‐equivalents were calculated in terms of the number of alternative treatments that could have been paid for using the same expenditure incurred differently. Resources such as beds are hence assumed to be monetised and the money spent elsewhere within healthcare.

Based on Equation (1), Approaches 1 and 2 can be written as:

(1.1) $${\mathrm{OC}}_{i}={{\mathrm{LOS}}_{i}}^{*}\frac{1}{{\mathrm{LOS}}_{j}}\;\left({}^{*}\mathrm{OCR}\right)$$

(1.2) $${\mathrm{OC}}_{i}={C_{i}}^{*}\frac{1}{C_{j}}$$

where *LOS_i_* is the incurred resource consumption of bed‐days, *LOS_j_* is the resource consumption of the forgone alternative patient, *OCR* is the (optional) occupancy rate of bed‐days, *C_i_* is the expenditure incurred, and *C_j_* is the expenditure of the forgone alternative use (e.g., treatments). For didactic reasons, we have separated fractions into two terms to demonstrate how to value the units consumed by patient *i*.

#### 3.2.2 Methodology B: Opportunity costs as the net benefit of the second‐best alternative forgone

The second methodology was used by four approaches that valued the units measured either monetarily (Approach 3) or in terms of the health benefit, usually QALYs, using patient‐equivalents (Approach 4) and/or local cost‐effectiveness thresholds (Approaches 5 and 6). Not all articles using patient‐equivalents reported them separately.

Monetary values took on providers' forgone gross expenditures, payment losses (mostly diagnosis‐related groups), or net revenue losses. One article adjusted the revenue in sensitivity analyses by 0–25% to account for an occupancy rate of 0.75–1.00 (Bayley et al., 2005).

The health benefit was usually expressed as the expected number of QALYs lost as a result of not being able to treat patients using the resources expended. For the patient‐equivalents forgone, one study derived QALY‐gain values from the published literature (Leroux, Morton, & Rivas, 2014). Another study recently aimed to estimate reimbursement tariffs by multiplying the marginal (gross) benefit with an assumed social value of a QALY of £50,000 (Kristensen, Siciliani, & Sutton, 2016). Others quantified the expected health benefit as the QALYs forgone of concurrently disinvested existing interventions (Oliver, 2002), or, more generally, by dividing the incurred expenditure by the monetary value assigned to health effects, taking as reference a conversion factor representing the cost‐effectiveness of marginal interventions paid for out of the same budget, that is, the local cost‐effectiveness threshold (Cookson, Drummond, & Weatherly, 2009; Coyle, Cheung, & Evans, 2014).

Based on Equation (2), Approaches 3(a–c), 4, 5, and 6 can be written as:

(2.1a) $${\mathrm{OC}}_{i}={{\mathrm{LOS}}_{i}}^{*}\frac{C_{j}}{{\mathrm{LOS}}_{j}}$$

(2.1b) $${\mathrm{OC}}_{i}={{\mathrm{LOS}}_{i}}^{*}\frac{R_{j}}{{\mathrm{LOS}}_{j}}\;\left({}^{*}\mathrm{OCR}\right)$$

(2.1c) $${\mathrm{OC}}_{i}={{\mathrm{LOS}}_{i}}^{*}\frac{\left(R_{j}-C_{j}\right)}{{\mathrm{LOS}}_{j}}$$

(2.2) $${\mathrm{OC}}_{i}={{\mathrm{LOS}}_{i}}^{*}\frac{B_{j}}{{\mathrm{LOS}}_{j}}$$

(2.3) $${\mathrm{OC}}_{i}={{\mathrm{LOS}}_{i}}^{*}\frac{{B_{j}}^{*}\lambda }{{\mathrm{LOS}}_{j}}$$

(2.4) $${\mathrm{OC}}_{i}={C_{i}}^{*}\frac{1}{\lambda }$$

where *R_j_* is the revenue of the forgone alternative patient, *B_j_* the health gain from treatment in terms of QALYs for the forgone alternative patient, and *λ* is the monetary value assigned to health effects as for example, expressed in local cost‐effectiveness thresholds.

#### 3.2.3 Methodology C: Opportunity costs as expenditure of the alternative chosen

The third methodology was used by two approaches, mainly differing in whether to multiply results (Approach 7) or present them separately (Approach 8).

The (health) economic convention of valuing the actually chosen alternative was widely followed by taking either market prices, national tariffs and payment sources, average expenditures from budgets and accounts, or reference costs. Stated‐preference techniques such as willingness‐to‐pay surveys were also used to elicit values.

Given that the opportunity costs of fixed resources such as bed‐days or clinic slots are not always adequately reflected by the monetary value of prices and payments, especially in the short run, they may be separated from the variable costs related to other consumables. In the short term, variable costs better indicate cost changes according to changes in resource consumption as no cash savings will be realised for the fixed costs proportion (Graves et al., 2007).

Based on Equation (3), Approaches 7 and 8 can then be written as:

(3.1) $${\mathrm{OC}}_{i}={{\mathrm{LOS}}_{i}}^{*}\frac{C_{i}}{{\mathrm{LOS}}_{i}}$$

(3.2) $${\mathrm{OC}}_{i}={\mathrm{LOS}}_{i}*\frac{{\mathrm{VC}}_{i}}{{\mathrm{LOS}}_{i}}\&{\mathrm{LOS}}_{i}$$

where *VC_i_* is the variable cost proportion of the expenditure incurred.

#### 3.2.4 Methodology D: Opportunity costs as the expenditure of the alternative chosen plus the highest net benefit forgone

The fourth methodology was used by one approach (Approach 9). Opportunity costs were represented as the (total) economic costs for providers, even though most articles identified opportunity costs as only the forgone net revenues; for instance: “The sum of opportunity cost and total cost defines the true cost of a surgical device” (Chatterjee, Chen, Goldenberg, Bae, & Finlayson, 2010, p.1076).

Based on Equation (4), Approach 9 can be written as:

(4.1) $${\mathrm{OC}}_{i}={{\mathrm{LOS}}_{i}}^{*}\left(\frac{C_{i}}{{\mathrm{LOS}}_{i}}+\frac{\left(R_{j}-C_{j}\right)}{{\mathrm{LOS}}_{j}}\right)$$

### 3.3 Illustrative comparison of approaches for valuing bed‐days

To highlight differences between the approaches, we present an illustrative example with three patients P_1_, P_2,_ and P_3_. To clarify the concept of making a choice between multiple alternative options, more than two patients are presented here following previous recommendations to avoid framing opportunity costs as binary decision problems (Ferraro & Taylor, 2005). Also, the second‐best use of bed‐days is assumed to not lie outside the healthcare sector.

Table 2 contains hypothetical values required for the illustration. Following an agent's objective of, for example, health or income maximisation, the known case mix is ranked as specifically as possible (e.g., on a ward level) to identify the patient with the highest net value. The existing approaches express the benefit as either monetary revenue or QALYs, which we chose to be the highest for patient P_1_ here; it is the optimally chosen patient *i*. Where applicable, this patient is compared to the second‐best alternative patient *j* (more precisely: patient group); in our example patient P_2_ and not P_3_. Note that the highest valued patient and the second‐best patient are unlikely to be each other's second‐best alternative; this is only true for the special case of identical marginal opportunity costs.

**Table 2:** Input data to illustrate the approaches to value opportunity costs

Occupancy rate	1.0^a^		
Cost‐effectiveness threshold (£)	20,000/QALY		
			
Patient(s)	P_1_	P_2_	P_3_
Units (bed‐days per patient)	10^b^	5	5
Expenditure (£ per patient)	7,000 (variable: 3,500)	5,000	5,000
Benefit (£ revenue per patient)	9,000	6,000	5,500
Benefit (QALY gain per patient)^c^	1.3	0.6	0.4

Note that all values are illustrative. Based on the highest (net) benefit expressed either monetarily or in QALYs, patient P_1_ is the optimally chosen patient *i*, patient P_2_ is the second‐best patient *j*, and patient P_3_ is the third‐best patient.

QALY = quality‐adjusted life year.

a Assumes full capacity and that freed beds are efficiently redeployed (Drummond et al., 2005),

b Excess consumption (not necessarily the total length of stay),

c Attributable to the treatment.

The value of the opportunity costs for the 10 bed‐days consumed by treating one patient *i* (i.e., what treating that patient is “worth”) according to the nine approaches is shown in Table 1. The results vary widely, even within the same methodology and when standardising the unit of outcome, suggesting that further appraisal of the approaches is needed.

### 3.4 Appraisal of existing approaches

The approach used to value bed‐days, and consequently the units in which their costs are expressed, must be chosen to match the decision‐making agent's objective(s). In our illustration based on the existing approaches, different agents such as providers, payers, or society may seek to maximise natural units, expenditures, revenues, net revenues, or health outcomes.

Approaches 1 and 2 lend themselves for analyses of natural units, for example, to maximise throughput. Expressing the relative costs of production as the exchange rate between natural units (Approach 1) reflects the true value of competing resource consumptions more accurately than the exchange rate between the expenditures associated with the natural units (Approach 2) given the independence of monetary inflation. In fact, if the expected exchange value differs from the expected cost value, a change in the optimal choice may occur (Buchanan, 1969). In our example, the expenditure of the 2 patient‐equivalents forgone is only valued as 1.4 treatment equivalents. Hence, an agent aiming to maximise throughput regards treating patient *i* less favourable, which likely distorts the bed‐days' true value.

Providers aiming to maximise income can readily calculate net benefits in the form of net revenues using Equation (2.1c) of Approach 3. The other equations of Approach 3, as well as Approaches 7 and 8, take merely the expenditure or payment, which makes the strong assumption of prices being at the equilibrium point of the perfectly competitive market model, requiring fulfilment of all conditions characterising such markets (Folland et al., 2010; Henry & Searles, 2012). This is unlikely given market imperfections in healthcare of, for example, quasi‐monopolies and price controls, information asymmetries, patents and licencing requirements, and non‐identical products (Arrow, 1963; Henry & Searles, 2012; Lave et al., 1994; Rattinger, Jain, Ju, & Mullins, 2008). Hence, spending £1 is unlikely to generate a benefit (revenue) equivalent to £1. This is illustrated in Table 2 for patients P_2_ and P_3_, for which we kept the bed‐day consumption and expenditures identical but varied the benefits, which determined P_2_ as second‐best patient *j* and P_3_ as third‐best patient. Approaches 7 and 8 additionally do not explicitly consider the second‐best alternative forgone. Approach 9 aims to correct for alternatives being in competitive disequilibrium, but requires identifying options as optimal and non‐optimal. It also does not correct for distortions of the optimal alternative's price, still producing flawed results then.

For payers and societies aiming to maximise health, health outcomes form the relevant benefit. The conversion of expenditures into QALYs (Approach 6) has been criticised for the missing link to actually displaced or unfunded services (Coyle et al., 2014). More importantly, Approaches 4, 5, and 6 do not calculate net benefits and may be less suitable for subsequent economic studies; more on this in the discussion. Hence, no approach currently exists that calculates net benefits for bed‐days using health outcomes.

### 3.5 Proposing an alternative to valuing bed‐days

Expressing the health benefit in monetary terms is exactly what is captured with the net monetary benefit, NMB (Claxton, 1999; Tambour, Zethraeus, & Johannesson, 1998), which is defined incrementally as:

(5) $$\mathrm{NMB}=\Delta B^{*}\lambda -\Delta C$$

where Δ*B* is the incremental benefit between two healthcare interventions, *λ* the monetary value per unit of health benefit gained as, for example, defined by a local cost‐effectiveness threshold, and Δ*C* the incremental expenditure between two healthcare interventions. Note that the NMB calculates the net benefit as required in Equation (2) of Methodology B. For our purposes, we exploit the idea of valuing health gains monetarily with conventional cost‐effectiveness thresholds as shown in Equation (5). Thus, decision makers aiming to maximise health can account for the net benefit of patient *j* forgone as follows:

(2.5) $${\mathrm{OC}}_{i}={{\mathrm{LOS}}_{i}}^{*}{\frac{\left({B_{j}}^{*}\lambda -C_{j}\right)}{{\mathrm{LOS}}_{j}}}^{*}\mathrm{OCR}$$

where *B_j_* is the marginal health gain from hospital treatment for the second‐best patient and *C_j_* denotes the marginal expenditure incurred on the hospital treatment of the second‐best patient, for payers thus the reimbursement payment. Note that the health benefit ought to account for the marginal gain of patients from treatment to avoid the contradictory conclusion that patients in no need of care will benefit the most from a hospital bed (e.g., a perfectly healthy individual with a utility score of 1), and to capture any changes in health occurring without the treatment; cf. discussion. In healthcare settings operating near full capacity and in the presence of an unmet demand from otherwise treated patients awaiting admission, the term OCR is to be omitted.

Applied to the data in Table 2, the opportunity costs of the bed‐days consumed by patient *i* equal £14,000 in terms of net benefits forgone for the second‐best patients *j*. By considering health maximisation as objective and the net monetary value of the forgone QALYs, this differs from the values calculated with the existing approaches of Methodology B, that is, providers' expenditures of £10,000, revenue of £12,000, net revenue of £2,000, gross monetary benefit of £24,000, and 1.2 gross QALYs forgone. Moreover, if the chosen alternative had not been optimal, Methodology D would need to be used.

(4.2) $${\mathrm{OC}}_{i}={{\mathrm{LOS}}_{i}}^{*}\left(\frac{C_{i}}{{\mathrm{LOS}}_{i}}+\frac{\left({B_{j}}^{*}\lambda -C_{j}\right)}{{\mathrm{LOS}}_{j}}\right)$$

However, P_1_ was the optimal choice as indicated in Table 3; it had the highest net benefit of all three alternatives, and only for P_1_ were the sum of the expenditure and the highest net benefit forgone smaller than the benefit incurred. Thus, the true value of the opportunity costs for the 10 bed‐days is here equal to the forgone second‐best net benefit of £14,000.

**Table 3:** Opportunity cost results of the 10 bed‐days used for patient P_1_ for decision makers aiming to maximise health

Patient(s)	P_1_ (*n* = 1)	P_2_ (*n* = 2 forgone)	P_3_ (*n* = 2 forgone)
Expenditure (£ in total)	**7,000**	**10,000**	10,000
Benefit (GMB, £ in total)	26,000	**24,000**	16,000
NMB (benefit‐expenditure, £ in total)	19,000	**14,000**	6,000
Expenditure + highest NMB forgone	**21,000**	29,000	29,000

*Note*. GMB = gross monetary benefit, NMB = net monetary benefit, QALY = quality‐adjusted life year.

Based on the highest (net) benefit expressed either monetarily or in QALYs, patient P_1_ is the optimally chosen patient *i*, patient P_2_ is the second‐best patient *j*, and patient P_3_ is the third‐best patient. Figures in bold correspond to approaches (and values) covered in the overview table.

## 4 DISCUSSION

This paper explored different estimation techniques for valuing the opportunity costs of resources. Although seen as fundamental in defining economics (Buchanan, 1969; O'Donnell, 2010; Robbins, 1932) and health economics (Drummond et al., 2005; Mushkin, 1958; Palmer & Raftery, 1999; Russell, 1992), the concept's underlying assumptions are frequently disregarded for reasons of pragmatism. As a result, the special case of perfect competition has become a widespread standard, despite it effectively disconnecting the choice problem from the mutually‐exclusive, second‐best use forgone. It is hence unsurprising to us that this definition made it into numerous economic textbooks as the convention for estimating opportunity costs (O'Donnell, 2010), fuelling the confusion of professional economists and others alike (Ferraro & Taylor, 2005).

When assigning cost values to resources, it is important for researchers to make their assumptions explicit, including on the aim, perspective, the associated consequences, and “any adjustments made to approximate to opportunity costs” (Husereau et al., 2013). Incorrectly attaching high cost values may give decision makers the illusion of potentially large cost savings of a programme that reduces resource consumption, and the actual cost savings may be more modest due to only being realisable on the variable cost components and if the freed fixed resources were almost immediately re‐deployed (Drummond et al., 2005; Graves et al., 2010). This is especially true for the short run, during which a high proportion of fixed costs will not change and hence should not influence the value of a particular choice from an economic viewpoint (Dawson, 1994; Graves et al., 2010).

As for the imperfectly‐marketed resource “bed‐days”, the existing approaches to costing produce results that differ widely. When using our framework to appraise the underlying assumptions, we revealed how approximations of the true value of bed‐days may be flawed and violate economic theory, depending on the chosen perspective. This includes the conventional use of reference costs, as the second‐best alternative is usually not explicitly accounted for. Moreover, average costs are unlikely to represent the marginal costs of producing one more unit (i.e., bed‐day) as the bulk of treatment costs can be expected to occur towards the beginning of a stay (Drummond et al., 2005). More generally, it can be questioned whether it is appropriate to adopt the ideas valid for the competitive market model to the imperfect healthcare market (Dawson, 1994). Nonetheless, many applications resorted to consider merely expenditures or payments. None of the existing approaches valued the health gain associated with bed‐days in terms of the net benefit. Consequently, we developed a novel approach using the net monetary benefit of second‐best patients, which we assert is most suitable for decision makers aiming to maximise health within a fixed healthcare budget, such as the National Health Service in the United Kingdom and its counterparts in other healthcare systems.

To value health outcomes monetarily, we follow the idea of using local cost‐effectiveness thresholds (Claxton, 1999; Tambour et al., 1998). Although controversial (Neumann, Cohen, & Weinstein, 2014), these thresholds are used in actual healthcare decision making (Drummond, 2013; Schwarzer et al., 2015). Such thresholds can also be estimated on the basis of displaced services at a system level either theoretically based on the decision maker's preference (Eckermann, 2015; Eckermann & Pekarsky, 2014) or empirically based on the opportunity costs of healthcare spending (Claxton et al., 2015). When assuming that expenditures not spent on bed‐days could be spent elsewhere in the healthcare system, which is the assumption behind reference costs, a general threshold should be used; otherwise a disease‐specific threshold may be more sensible when beds saved have to be filled by alternative patients within the same specialty or setting.

As an alternative to the NMB one could use the net health benefit, NHB (Stinnett & Mullahy, 1998); see Table 1 for the additional equations. Although illustrative to quantify the expected health benefits forgone, the outcome is then converted to units such as QALYs, which do not have an equivalent expression for other resources and outside the health sector. Hence, we prefer the NMB as in Equation (2.5), whose monetary units also underline its character of being an alternative to conventional methods. Likewise, next to patient‐equivalents one could resort to treatment‐equivalents, bearing in mind that expenditures encounter monetary inflation and may result in distorted bed‐day values (cf. Section 3.4).

Ideally, our approach is used by observing actually displaced treatments or patients in a particular setting. For instance, if Table 2 showed three actual patients, not patient groups, the 10 bed‐days consumed by P_1_ could have been used to treat both displaced patients P_2_ and P_3_ as each consumed 5 bed‐days. However, we acknowledge that such observations are not always practical or indeed possible, for example, when actual patients do not even present at a healthcare facility given their pre‐existing knowledge that they cannot be treated there (e.g., for capacity constraints). To enhance generalisability, a pragmatic compromise may be to use patient groups from the regular patient population awaiting admission to a particular ward. Potential heterogeneity of patients should be taken into account by, for example, considering patient sub‐groups. From a payer perspective, specific reimbursement payments may be taken as expenditure for the different patient groups, possibly adjusted for price distortions such as subsidies (Drummond et al., 2005) or excessively high taxes (Donaldson, 1990). In settings with local cost‐effectiveness thresholds, the marginal length of stay, expenditure, and health benefit of the forgone patients then need to be determined. These could, for example, be approximated context‐specifically with an average from national or local studies. Note that from the healthcare payer perspective, the marginal expenditure and length of stay are equivalent to the actual reimbursement payment and length of stay given that without hospitalisation, there is no hospital treatment to pay for nor will patients have consumed any hospital resources. Conversely, the marginal health gain of patients from a particular hospital treatment will depend on the condition analysed in order to account for changes in health without treatment, including for example, death for otherwise fatal conditions if not medically attended in hospital, and the alleviation and prevention of symptoms or disease for non‐life‐threatening conditions. Health benefit estimations could be based on the natural history of diseases, representative health data such as disability weights from the Global Burden of Disease Study (Salomon et al., 2015) or quality‐of‐life scores for diseased and healthy populations (Claxton et al., 2015), and potentially even standard care for conditions for which it would be impracticable or unethical to determine the natural course; see the Appendix for two illustrations of such implementations.

Special consideration may be needed for temporary decreases in supply and increases in demand for bed‐days following exogenous shocks (i.e., unplanned events) such as infectious disease epidemics (Plowman et al., 2001), heat waves, cold weather, natural disasters, strikes of healthcare personnel, or sudden reductions in funding. For example, for infectious disease outbreaks calculations of total length‐of‐stay may be subject to time‐dependent biases with only a proportion being an “excess” stay (Graves et al., 2010), and the isolation or closure of bays and wards may lead to additional bed‐days lost unoccupied. Altogether, all beds lost attributable to the exogenous shock should be added to the number of bed‐days consumed by treating patients during an avoidable event. Adjusting bed occupancy to account for capacity should only be done in situations where there is no excess demand (i.e., no shortages or waiting lists). No opportunity costs will then be attached to the empty beds in terms of health forgone for other patients, and other than the cash value of reducing the idle capacity in the long‐term (with associated consequences on marginal expenditures and revenues). However, full capacity does not mean all beds being occupied; besides, there may be other limiting factors than the occupancy rate, for example, the theatre use rate for surgical patients.

### 4.1 Strengths and limitations

We based our taxonomy of methodologies on extensive reviews of the concept of opportunity costs, including 22 well‐known economic textbooks (O'Donnell, 2010) as well as James Buchanan's comprehensive treatise (Buchanan, 1969). Existing applications to value bed‐days were searched for in a scoping review due to our aim and the paucity of relevant and consistently used search terms. Included articles covered a wide range of journals, interventions, and disease areas (see Appendix), which may point towards the general relevance of an interest in the topic among healthcare professions, as well as the broad applicability for our novel approach.

Although we did not explicitly consider indirect costs using wage rates as proxy for opportunity costs, these applications are addressed in Approach 3 as the time loss is simply multiplied with a wage rate. Also, in reporting the results of our review, we did not state the number of articles or applications of each approach. Our intent was to focus on the differences per approach and not on the number of applications per approach, which in any case may be biased towards those that are practical given the data available to authors rather than those based on sound theoretical methodology.

Focusing on “bed‐days” has advantages as it is a broadly used resource across indications, a major cost driver and hence influential for all kinds of analyses involving patient stays, relatively easy to measure through “length of stay”, and universally used (across countries, time and settings such as hospitals, long‐term care, and mental health facilities). Additionally, the opportunity costs of bed‐days lost due to isolation or closure of wards can be calculated on the same scale as those directly consumed by patients. By identifying bed‐days as resources, this paper concentrated on the last two steps of costing of identification, measurement, and valuation (Drummond et al., 2005; Mogyorosy & Smith, 2005). As such, our approach anchors on the identified resource itself as the most important unit to measure costs. Then continuing to value the resources with the marginal net benefit is important to account for the different health benefits and expenditures associated with alternative options (cf. Methodology B), although a disaggregated presentation may facilitate a broader and more explicit multi‐criteria decision analysis framework, for example, when thinking about the value of reducing waiting lists or when incorporating non‐pecuniary decision factors such as emotional distress. Moreover, one could also try to elicit the preferences for bed‐days to patients and providers; see, for example, (Stewardson, Harbarth, Graves, & TIMBER Study Group, 2014) for a study on the willingness‐to‐pay of 11 hospital administrators.

Despite our focus on bed‐days, the concepts presented in this paper can be readily adapted to other goods and resources used to treat patients, such as operating theatre slots or time spent in a general practitioner clinic, as well as to evaluations with other economic perspectives and optimisation objectives. As our illustration was based on existing applications, it considered either health, throughput, or (net) revenue maximisation but not equity concerns.

Although our approach values opportunity costs with the NMB, it is not used incrementally here to evaluate two or more different healthcare interventions (Drummond et al., 2005). A full economic evaluation requires additional input parameters for different interventions, including at least their expenditures and effectiveness. Also note that we consider a broader perspective for the opportunity costs across indications (e.g., the second‐best alternative patient for specific wards), and economic evaluations typically compare a new intervention to standard care within indications (which is presumed to be the second‐best alternative treatment for specific diseases).

Lastly, adequate costing may be quite complex but attempting to identify the second‐best patients improves the existing pragmatic convention as it enhances applied research and decision‐making by coming closer to the theoretical ideal of how to estimate the true costs of resources. Nonetheless, the confidence one has in the opportunity cost estimates will depend largely on how well the actual second‐best use has been defined and measured.

## 5 CONCLUSION

This paper has highlighted an underrated issue in costing whose consequences are often not apparent to decision makers, and which has been shown to confuse professional economists too. To summarise, opportunity costs are inherently linked to choice in economics and the net trade‐off cost of the second‐best use forgone.

Bearing these theoretical considerations in mind, when aiming to estimate the opportunity costs of resources adequately, is crucial for sound economic research and decision making. Various methodologies were developed in the past that rely on different assumptions. When using the taxonomic framework that we developed to appraise the underlying assumptions, we found that opportunity costs are often applied to valuing bed‐days in ways that are flawed and violate economic theory. For pragmatic reasons, the special case of perfect competition has become a convention for estimating opportunity costs, as is demonstrated through the common use of reference costs and administrative accounting data. By relying solely on the incurred resource consumption, cost factors are effectively disconnected from choice and the second‐best alternative use forgone. Researchers should (a) be aware of the underlying assumptions and resulting biases when applying these approaches, which frequently remain unmentioned and unquestioned; (b) carefully consider the adequate costing approach dependent on an agent's objective and perspective; and (c) explicitly state any potential implications in a manner that is comprehensible to decision makers. For decision makers aiming to maximise health, we propose a novel alternative for bed‐days that effectively re‐connects to the concept of choice and explicitly considers net benefits.

## CONFLICT OF INTEREST

The authors have no conflict of interest.

## Supporting information

Table A1: Search syntaxes used for the scoping literature review of existing applications suitable to estimate the opportunity costs of bed‐days (last search on 02 December 2016).
Figure A1. Flowchart of the scoping literature review of existing applications suitable to estimate the opportunity costs of bed‐days.
Table A2: Overview of relevant papers detailing existing applications suitable to estimate the opportunity costs of bed‐days.
Table A3. Input data to illustrate the approaches to value the opportunity costs: gastroenteritis cases
Table A4. Overview of approaches to value the opportunity costs of bed‐days used for patients with acute gastroenteritis
Table A5. Opportunity cost results of the 80 bed‐days used for gastroenteritis cases using Approach New5.
Table A6. Input data to illustrate the value of the opportunity costs: TLH in terms of TAH.
Table A7. Overview of approaches to value the opportunity costs of bed‐days for TLH surgery
Table A8. Opportunity cost results of the 3,760 bed‐days used for TLH procedures using Approach New1.
