# Supplementary material for: Estimating the opportunity costs of bed‐days
Source: Health Econ. 2017 Nov 6;27(3):592–605. doi: 10.1002/hec.3613 (PMC5900745; doi:10.1002/hec.3613)
Supplement: Supplementary file 1 — Table A1: Search syntaxes used for the scoping literature review of existing applications suitable to estimate the opportunity costs of bed‐days (last search on 02 December 2016). Figure A1. Flowchart of the scoping literature review of existing applications suitable to estimate the opportunity costs of bed‐days. Table A2: Overview of relevant papers detailing existing applications suitable to estimate the opportunity costs of bed‐days. Table A3. Input data to illustrate the approaches to value the opportunity costs: gastroenteritis cases Table A4. Overview of approaches to value the opportunity costs of bed‐days used for patients with acute gastroenteritis Table A5. Opportunity cost results of the 80 bed‐days used for gastroenteritis cases using Approach New5. Table A6. Input data to illustrate the value of the opportunity costs: TLH in terms of TAH. Table A7. Overview of approaches to value the opportunity costs of bed‐days for TLH surgery Table A8. Opportunity cost results of the 3,760 bed‐days used for TLH procedures using Approach New1. [file HEC-27-592-s001.docx]

## Appendix

| Table A1: Search syntaxes used for the scoping literature review of existing applications suitable to estimate the opportunity costs of bed-days (last search on 02^nd^ December 2016). | | |
| --- | --- | --- |
| PubMed (NLM) | | |
| # | Searches | Records |
| 1 | (“bed day” OR “bed days”) AND (cost OR costs) | 701 |
| 2 | (“bed day” OR “bed days”) AND demand* | 90 |
| 3 | (“bed day” OR “bed days”) AND valu* | 139 |
| 4 | (“opportunity cost” OR “opportunity costs”) AND (“bed day” OR “bed days”) | 6 |
| 5 | (“opportunity cost” OR “opportunity costs”) AND health* | 808 |
| 6 | (“opportunity cost” OR “opportunity costs”) AND hospital | 254 |
| EconLit (Ovid) | | |
| # | Searches | Records |
| 1 | (bed day* and cost*).af. | 10 |
| 2 | (bed day* and demand*).af. | 1 |
| 3 | (bed day* and valu*).af. | 2 |
| 4 | (opportunity cost* and bed day*).af. | 0 |
| 5 | (opportunity cost* and health*).af. | 239 |
| 6 | (opportunity cost* and hospital*).af. | 23 |
| NLM: National Library of Medicine | | |

Figure A1. Flowchart of the scoping literature review of existing applications suitable to estimate the opportunity costs of bed-days.

Additional records identified through other sources

**n = 10**

PubMed (NLM) = 1,998

EconLit (Ovid) = 275

**n = 2,273**

Records identified

**n = 2,283**

Duplicate records excluded

**n = 424**

Title/abstract screened

**n = 1,859**

Records excluded

- No opportunity costs = 969
- No opportunity costs approach = 156
- No suitable approach for bed-days = 89
- Focussing on time with wage = 224
- Not in English = 79

**n = 1,517**

Records excluded

- No opportunity costs approach = 159
- No suitable approach for bed-days = 37
- Focussing on time with wage = 38
- Not in English = 1
- Merely abstract/presentation = 2
- No access (through British Library) = 4

**n = 241**

Relevant articles

**n = 101**

Full-text articles assessed for eligibility

**n = 342**

| Table A2: Overview of relevant papers detailing existing applications suitable to estimate the opportunity costs of bed-days. | | |
| --- | --- | --- |
| Approach | Description | Records |
| *Methodology A: Units of the second-best alternative forgone* | | |
| 1 | Patient-equivalents (of second-best patients *j*) forgone | ([Connell et al., 2015](#_ENREF_17); [Coughlan & O'Neill, 2001](#_ENREF_19); [Eddy, 1994](#_ENREF_25); [Falvo et al., 2007](#_ENREF_28); [Greenspoon et al., 2012](#_ENREF_41); [Harper, 1979](#_ENREF_44); [Herzer, Niessen, Constenla, Ward, & Pronovost, 2014](#_ENREF_46); [Huynh, Kleerup, Raj, & Wenger, 2014](#_ENREF_54); [Leroux, Morton, & Rivas, 2014](#_ENREF_61); [Lucas et al., 2009](#_ENREF_64); [Luce & Elixhauser, 1990](#_ENREF_65); [Mayhew, Ickx, Newbrander, Stanekzai, & Alawi, 2015](#_ENREF_69); [Navarro & Kaplan, 1996](#_ENREF_75); [Nicks & Manthey, 2012](#_ENREF_77)) |
| 2 | Treatment-equivalents forgone for the second-best patients *j* | ([Coyle, Cheung, & Evans, 2014](#_ENREF_20); [Eddy, 1994](#_ENREF_25); [Hollinghurst, Kessler, Peters, & Gunnell, 2005](#_ENREF_49); [Navarro & Kaplan, 1996](#_ENREF_75); [Pashayan, Lyratzopoulos, & Mathur, 2006](#_ENREF_82); [van de Vooren, Curto, & Garattini, 2014](#_ENREF_103)) |
| *Methodology B: Net benefit of the second-best alternative forgone* | | |
| *Valuation in terms of money* | | |
| 3 | Monetary value forgone on the second-best patient-equivalents | ([Aitken, 2012](#_ENREF_3); [Ang, Sabharwal, Johannsson, Bhattacharya, & Gupte, 2016](#_ENREF_4); [Basu, Phillips, Bitton, Song, & Landon, 2015](#_ENREF_7); [Bayley et al., 2005](#_ENREF_9); [Falvo et al., 2007](#_ENREF_28); [F. Fuertes-Guiro & Girabent-Farres, 2016](#_ENREF_29); [Fernando Fuertes-Guiro, Girabent-Farres, & Viteri-Velasco, 2016](#_ENREF_30); [Gammon & Lizotte, 2004](#_ENREF_32); [Herzer et al., 2014](#_ENREF_46); [Jenks, Laurent, McQuarry, & Watkins, 2014](#_ENREF_56); [Lucas et al., 2009](#_ENREF_64); [Mahajan et al., 2007](#_ENREF_67); [Mangan et al., 1992](#_ENREF_68); [Nicks & Manthey, 2012](#_ENREF_77); [Rojo-Manaute et al., 2012](#_ENREF_88); [Sasor, Flores, Wooden, & Tholpady, 2013](#_ENREF_94); [Southey et al., 2015](#_ENREF_95); [Wagner et al., 2014](#_ENREF_104)) |
| 5 | Gross monetary benefit forgone for the second-best patient-equivalents | ([Kristensen, Siciliani, & Sutton, 2016](#_ENREF_58)) |
| *Valuation in terms of health benefit (typically QALYs)* | | |
| 4 | Gross health benefit forgone for second-best patient-equivalents | ([Leroux et al., 2014](#_ENREF_61)) |
| 6 | Health benefit forgone for expected second-best use | ([Cookson, Drummond, & Weatherly, 2009](#_ENREF_18); [Coyle et al., 2014](#_ENREF_20); [Holdefer & Skinner, 2016](#_ENREF_48); [Oliver, 2002](#_ENREF_80); [Russell, 1992](#_ENREF_90)) |
| *Methodology C: Expenditure of the alternative chosen* | | |
| **7** | Expenditure for the resource consumption incurred | ([Abramovitch, Newman, Padaliya, Gill, & Charles, 2005](#_ENREF_1); [Adam, Evans, & Murray, 2003](#_ENREF_2); [Ang et al., 2016](#_ENREF_4); [Banks, Graves, Bauer, & Ash, 2010](#_ENREF_5), [2013](#_ENREF_6); [Baugh & Bohan, 2008](#_ENREF_8); [Bayley et al., 2005](#_ENREF_9); [Binney, Quest, Feingold, Buchman, & Majesko, 2014](#_ENREF_10); [Bodanapally et al., 2013](#_ENREF_11); [Buist et al., 2014](#_ENREF_12); [Chatterjee, Chen, Goldenberg, Bae, & Finlayson, 2010](#_ENREF_13); [Chatterjee, McCarthy, Montagne, Leong, & Kerrigan, 2011](#_ENREF_14); [Chatterjee, Payette, Demas, & Finlayson, 2009](#_ENREF_15); [Chinai et al., 2013](#_ENREF_16); [Danial et al., 2016](#_ENREF_21); [Ehreth, 1996](#_ENREF_26); [Falcone & Feinn, 2016](#_ENREF_27); [Furber & Donaldson, 1992](#_ENREF_31); [Gammon & Lizotte, 2004](#_ENREF_32); [Graves, 2004](#_ENREF_34); [Graves, Birrell, & Whitby, 2005](#_ENREF_35); [Graves et al., 2008](#_ENREF_36); [Graves et al., 2010](#_ENREF_37); [Graves et al., 2013](#_ENREF_38); [Graves, Nicholls, & Morris, 2003](#_ENREF_39); [Gruen, Chang, & MacLellan, 1996](#_ENREF_42); [Halton, Cook, Paterson, Safdar, & Graves, 2010](#_ENREF_43); [Hsu, 2011](#_ENREF_51); [Hubner, Hubner, Hopert, Maletzki, & Flessa, 2014](#_ENREF_52); [Hubner et al., 2015](#_ENREF_53); [Israelsson & Wimo, 2000](#_ENREF_55); [Kilgore, Steindel, & Smith, 1999](#_ENREF_57); [Lave et al., 1994](#_ENREF_59); [Lee et al., 2011](#_ENREF_60); [Lewis & Asch, 1999](#_ENREF_62); [Lopman et al., 2004](#_ENREF_63); [Lucioni, Mazzi, & Rossi, 2005](#_ENREF_66); [Mangan et al., 1992](#_ENREF_68); [McKay, 2013](#_ENREF_71); [Mutters, Gunther, Frank, & Mischnik, 2016](#_ENREF_73); [Nguyen, Chaboyer, & Whitty, 2015](#_ENREF_76); [O'Brien & Donato, 1993](#_ENREF_79); [Otter et al., 2016](#_ENREF_81); [Peeraully, Henderson, & Davies, 2016](#_ENREF_83); [Perroca, Jerico Mde, & Facundin, 2007](#_ENREF_84); [Piednoir et al., 2010](#_ENREF_85); [Rodney, Hardison, Rodney-Arnold, & McKenzie, 2006](#_ENREF_87); [Rosemurgy et al., 2016](#_ENREF_89); [Santillan et al., 2008](#_ENREF_93); [Spencer, Coast, Spry, Smith, & Sparrow, 1995](#_ENREF_96); [Taheri et al., 1998](#_ENREF_98); [Turner-Stokes, 2007](#_ENREF_99); [Turner-Stokes, Bill, & Dredge, 2012](#_ENREF_100); [Turner-Stokes, Poppleton, Williams, Schoewenaars, & Badwan, 2012](#_ENREF_101); [Turner-Stokes, Sutch, & Dredge, 2012](#_ENREF_102); [Ward, Eckman, Schauer, Raja, & Collins, 2011](#_ENREF_105); [Wariyapola et al., 2016](#_ENREF_106); [Young et al., 2010](#_ENREF_107)) ([Desaigues, 2001](#_ENREF_23); [Gill, Griffin, & Hesketh, 2013](#_ENREF_33); [Hjalte, Norinder, Persson, & Maraste, 2003](#_ENREF_47); [Mentzakis, Ryan, & McNamee, 2011](#_ENREF_72); [Richardson, Iezzi, Sinha, Khan, & McKie, 2014](#_ENREF_86); [Stewardson, Harbarth, Graves, & Timber Study Group, 2014](#_ENREF_97)) |
| 8 | Separating variable expenditure and non-monetary resource consumption | ([Graves et al., 2007](#_ENREF_40); [Halton et al., 2010](#_ENREF_43); [McIntosh, Donaldson, & Ryan, 1999](#_ENREF_70)) |
| *Methodology D: Expenditure of the alternative chosen + highest net benefit forgone* | | |
| 9 | Expenditure incurred + highest net revenue forgone | ([Baugh & Bohan, 2008](#_ENREF_8); [Chatterjee et al., 2010](#_ENREF_13); [Chatterjee et al., 2011](#_ENREF_14); [Chatterjee et al., 2009](#_ENREF_15); [Hsiao & Stason, 1979](#_ENREF_50)) |
| QALY: quality-adjusted life year | | |

**Two real-life examples to illustrate the opportunity cost estimation.**

The following two examples illustrate how implementation of the approaches we outline could look like in practice. We have made some simplifying assumptions to ensure clarity.

**Example 1: Value of bed-days used for patients with acute gastroenteritis in England.**

Hospital admission of otherwise completely healthy individuals for an episode of acute gastroenteritis is discouraged in England due to the self-limiting nature of symptoms, the limited treatment options, and the risk of admitted patients causing outbreaks of infectious intestinal disease potentially leading to severe service disruptions ([Norovirus Working Party, 2012](#_ENREF_78)). If for instance 40 patients with acute gastroenteritis stayed each 2 days in an acute hospital, the excess resource consumption would have been 80 bed-days. The mean costs for gastroenteritis cases staying in hospital in England in 2015/16 was £1,594 per patient according to the average standard costs list of the National Health Service (NHS) ([Department of Health, 2016](#_ENREF_22)). In order to be able to determine whether treating the gastroenteritis cases in hospital was the optimal alternative, cf. Methodology D, we also need information on their health benefit gained from the treatment, which we operationalised as the reduction of disutility using the Global Burden of Disease Study: The disability weights for mild, moderate and severe diarrhoea were 0.074, 0.188 and 0.247 ([Salomon et al., 2015](#_ENREF_92)). If hospital treatment led to alleviating the gastrointestinal distress symptoms of patients from severe to mild, there would be a gain in health benefit for the patients for the 2 days of experiencing symptoms of (0.247−0.074)/365*2=0.001 (the length of stay may also be reduced when shortening the duration of a disease, which we ignore here for simplicity).

Turning to the second-best patients forgone, we first assume that their characteristics can be approximated with the average of those for the regularly admitted non-gastroenteritis patient population. Their length-of-stay (LOS) is assumed to be the mean hospital LOS of patients in England in 2015/16 of 5 days ([Health and Social Care Information Centre, 2016](#_ENREF_45)), while the mean costs for all non-gastroenteritis cases was £2,627 per patients ([Department of Health, 2016](#_ENREF_22)). In addition, we assume a hypothetical hospital revenue (i.e. payments from healthcare payers, i.e. local authorities, to the hospital) of 10% of the expenditure; i.e. £2,627*1.1=£2,890. On average, inpatients will gain much more in terms of health restored and/or maintained when treated for other conditions than acute gastroenteritis. For instance, the disability weight for the most severe forms of stroke with long-term consequences plus cognition problems is 0.588, while the least severe forms of a mild stroke with long-term consequences is 0.019 ([Salomon et al., 2015](#_ENREF_92)). In case timely accessing a free hospital bed for treatment thus prevents or alleviates the most severe forms of a stroke, there would be a potential health benefit gain of 0.588−0.019=0.569; cf. the estimated 0.472 QALYs gained per patient over ten years for acute stroke unit care vs. standard care in a general medical ward ([Saka, Serra, Samyshkin, McGuire, & Wolfe, 2009](#_ENREF_91)). Even higher health gains may be achievable for patients with potentially fatal conditions if they were to die without treatment. For instance, acute myocardial infarction is associated with disability weights of 0.432 on day 1-2 and 0.074 on day 3-28 ([Salomon et al., 2015](#_ENREF_92)); if patients thus were to die without treatment but otherwise survived and lived healthy for at least one year, the health gain would be equivalent to (1−0.432)/365*2 + (1−0.074)/365*26 + (1−0)/365*(365−2−26) = 0.992. Clinicians may rightfully argue that there will always be a bed made available for emergency patients, or that a constant flow of acute myocardial infarctions will not present themselves even at the largest of emergency departments. Therefore, we continue this illustration by taking the exemplary health gain of the stroke patients of 0.569.

Furthermore, we assumed full capacity since most NHS hospitals in England operate at full capacity and face waiting lists. We took as monetary value for the health benefit gained the reference case of the local cost-effectiveness threshold of £20,000/QALY for England and Wales ([National Institute for Health and Care Excellence, 2013](#_ENREF_74)). All inputs are shown in Table A3 below, based on Table II in the paper:

| Table A3. Input data to illustrate the approaches to value the opportunity costs: gastroenteritis cases | | |
| --- | --- | --- |
| Occupancy rate | 1.0^a^ |  |
| Cost-effectiveness threshold (£ per QALY) | 20,000^b^ |  |
|  |  |  |
| Patient(s) | P_1_ (acute gastroenteritis cases, n=40) | P_2_ (regularly admitted, non-gastroenteritis patient) |
| Units (bed-days per patient) | 2 (total for 40 patients=80) | 5^e^ |
| Expenditure (£ per patient) | 1,594 (variable: 239)^c^ | 2,627^f^ |
| Revenue (£ per patient) | - | 2,890^g^ |
| Benefit (QALY gain per patient) | 0.001^d^ | 0.569^h^ |
| NHS: National Health Service, NICE: National Institute for Health and Care Excellence, QALY: quality-adjusted life year.  a: assumes full capacity and that freed beds are efficiently redeployed ([Drummond, Sculpher, Torrance, O'Brien, & Stoddart, 2005](#_ENREF_24)).  b: local cost-effectiveness threshold value of the reference case in England and Wales ([National Institute for Health and Care Excellence, 2013](#_ENREF_74)).  c: Mean NHS reference costs of gastroenteritis cases staying in hospital in England in 2015/16, activity-weighted ([Department of Health, 2016](#_ENREF_22)).  d: Hypothetical mean health benefit gain from hospital treatment for gastroenteritis cases based on disability weights for diarrhoea and an alleviation of a potentially severe to a mild presentation (Salomon et al., 2015).  e: Mean hospital length of stay of patients in England in 2015/16 ([Health and Social Care Information Centre, 2016](#_ENREF_45)).  f: Mean NHS reference costs of non-gastroenteritis cases staying in hospital in England in 2015/16, activity-weighted, ([Department of Health, 2016](#_ENREF_22)).  g: Mean revenue of hypothetical 10% of the expenditure.  h: Hypothetical mean health benefit gain from hospital treatment for non-gastroenteritis cases based on disability weights for stroke and an alleviation of a potentially severe to a mild presentation (Salomon et al., 2015). | | |

Based on this information, the direct expenditure incurred by the gastroenteritis cases was £63,760 (approach 7). When assuming that variable costs make up for 15% of the total costs, preventing the outbreak would have resulted in cash savings of £9,564 (approach 8).

In terms of displaced alternatives, the value of the 80 bed-days is equivalent to having forgone 16 non-gastroenteritis patients (approach 1), 24.3 treatments for the non-gastroenteritis patients (approach 2), a QALY gain between 3.2 to 10.6 (approach 6 and New4), and a monetary value ranging from a forgone hospital profit of £4,203 (approach 3c) to a forgone net monetary benefit for the treatment-equivalents of £212,444 (approach New2); for details see Table A4.

| Table A4. Overview of approaches to value the opportunity costs of bed-days used for patients with acute gastroenteritis | | | |
| --- | --- | --- | --- |
| Approach | Description | Equation | Results for patient *i* |
| *Methodology A: Units of the second-best alternative forgone* | | | |
| 1 | Patient-equivalents (of non-gastroenteritis patients *j*) forgone | ${LOS}_{i}*\frac{1}{{LOS}_{j}} \left( *OCR \right)$ | 16.0 |
| 2 | Treatment-equivalents forgone for the non-gastroenteritis patients *j* | $C_{i}*\frac{1}{C_{j}}$ | 24.3 |
| *Methodology B: Net benefit of the second-best alternative forgone* | | | |
|  | *Valuation in terms of money* |  |  |
| 3a | Expenditure forgone on the non-gastroenteritis patient-equivalents | ${LOS}_{i}*\frac{C_{j}}{{LOS}_{j}}$ | £42,032 |
| 3b | Revenue forgone from the non-gastroenteritis patient-equivalents | ${LOS}_{i}*\frac{R_{j}}{{LOS}_{j}} \left( *OCR \right)$ | £46,235 |
| 3c | Net revenue forgone from the non-gastroenteritis patient-equivalents | ${LOS}_{i}*\frac{\left( R_{j}-C_{j} \right)}{{LOS}_{j}}$ | £4,203 |
| 5 | Gross monetary benefit forgone for the non-gastroenteritis patient-equivalents | ${LOS}_{i}*\frac{\left( B_{j}*\lambda\right)}{{LOS}_{j}}$ | £182,080 |
| New1 | Net monetary benefit forgone for the non-gastroenteritis patient-equivalents | ${LOS}_{i}*\frac{\left( B_{j}*\lambda-C_{j} \right)}{{LOS}_{j}}$ | £140,048 |
| New2 | Net monetary benefit forgone for the non-gastroenteritis treatment-equivalents | $C_{i}*\frac{\left( B_{j}*\lambda-C_{j} \right)}{C_{j}}$ | £212,444 |
|  | *Valuation in terms of health benefit (typically QALYs)* |  |  |
| 4 | Gross health benefit forgone for non-gastroenteritis patient-equivalents | ${LOS}_{i}*\frac{B_{j}}{{LOS}_{j}}$ | 9.1 |
| 6 | Health benefit forgone for expected second-best use | $C_{i}*\frac{1}{\lambda}$ | 3.2 |
| New3 | Net health benefit forgone for the non-gastroenteritis patient-equivalents | ${LOS}_{i}*\frac{\left( B_{j}-\left( \frac{C_{j}}{\lambda} \right) \right)}{{LOS}_{j}}$ | 7.0 |
| New4 | Net health benefit forgone for the non-gastroenteritis treatment-equivalents | $C_{i}*\frac{\left( B_{j}-\left( \frac{C_{j}}{\lambda} \right) \right)}{C_{j}}$ | 10.6 |
| *Methodology C: Expenditure of the alternative chosen* | | | |
| 7 | Expenditure for the resource consumption incurred | ${LOS}_{i}*\frac{C_{i}}{{LOS}_{i}}$ | £63,760 |
| 8 | Separating variable expenditure and non-monetary resource consumption | ${{LOS}_{i}*\frac{{VC}_{i}}{{LOS}_{i}} \& LOS}_{i}$ | £9,564 & 80 |
| *Methodology D: Expenditure of the alternative chosen + highest net benefit forgone* | | | |
| 9 | Expenditure incurred + highest net revenue forgone | ${LOS}_{i}*\left( \frac{C_{i}}{{LOS}_{i}}+\frac{\left( R_{j}-C_{j} \right)}{{LOS}_{j}} \right)$ | £67,963 |
| New5 | Expenditure incurred + highest net monetary benefit forgone | ${LOS}_{i}*\left( \frac{C_{i}}{{LOS}_{i}}+\frac{\left( B_{j}*\lambda-C_{j} \right)}{{LOS}_{j}} \right)$ | £203,808 |
| The last column illustrates the marginal opportunity costs of gastroenteritis patients *i* consuming 80 bed-days.  B: (health) benefit gained per patient, C_i_: total expenditure incurred for *i*, C_j_: expenditure incurred per patient, λ: monetary value assigned to QALYs in local cost-effectiveness thresholds, LOS_i_: total bed-day consumption of *i*, LOS_j_: length of stay per patient, OCR: occupancy rate, QALY: quality-adjusted life year, R: revenue per patient, VC: variable cost proportion of the expenditure. | | | |

When comparing the conventional NHS reference costs to the net monetary benefit forgone for the second-best patients, this illustrative example shows a difference of £63,760 (approach 7) vs. £140,048 (approach New1), which is more than twice the value of the bed-days used for the gastroenteritis cases. This difference is largely driven by the forgone QALY gain and the monetary value assigned to QALYs; e.g. when using the upper-bound cost-effectiveness threshold of NICE of £30,000/QALY, the forgone net monetary benefit resulted in £231,088 or almost four times the figure with the conventional approach 7.

While it may sound plausible that treating gastroenteritis cases is not the optimal choice for bed occupancy, one can investigate this more formally by comparing the net benefits achievable; the highest one determines the optimal choice. For decision makers aiming to maximise health, we already know the net monetary benefit of the forgone non-gastroenteritis patients of £140,048 (cf. approach New1); we still need to calculate the net monetary benefit for the gastroenteritis cases (i.e., benefit minus expenditure): 40*(0.001*£20,000) − £63,760 = £800 − £63,760 = -£62,960 (an economic loss); see Table A5 below. Thus, the higher net benefit would have been achieved with the non-gastroenteritis cases, which renders the gastroenteritis cases a sub-optimal alternative. Consequently, methodology D becomes the adequate estimation technique to apply for health-maximising decision makers, with the value of the opportunity costs of the 80 bed-days used for the gastroenteritis cases being actually equivalent to £203,808 (approach New5), providing a strong economic argument for ideally avoiding (or reducing) these hospital stays due to acute gastroenteritis.

| Table A5. Opportunity cost results of the 80 bed-days used for gastroenteritis cases using Approach New5. | | |
| --- | --- | --- |
| Patient(s) | P_1_ (acute gastroenteritis cases, n=40) | P_2_ (forgone regularly admitted, non-gastroenteritis patients, n=16) |
| Expenditure (£ in total) | **63,760** | **42,032** |
| Benefit (GMB, £ in total) | 800 | **182,080** |
| NMB (benefit-expenditure, £ in total) | -62,960 | **140,048** |
| Expenditure + highest NMB forgone | **203,808** | -20,928 |
| GMB: gross monetary benefit, NMB: net monetary benefit, QALY: quality-adjusted life year. Figures in **bold** correspond to approaches covered in our overview table.  The NMB is higher for P_2_ than for P_1_, and the “Expenditure + highest NMB forgone” is smaller than the “Benefit (GMB, £ in total)” for P_2_ while it is higher for P_1_. Everything indicates to that P_1_ is the sub-optimal alternative, and the value of the opportunity costs for the 80 bed-days is the “Expenditure + highest NMB forgone” of £203,808. | | |

**Example 2: Value of bed-days used for competing surgical procedures in Australia.**

In contrast to example 1 where we looked at different patients (i.e. cases and displaced alternative patients), example 2 estimates the value of the bed-days used for two competing procedures in the same patients, thus forgoing the use of bed-days for treating patients with one procedure in favour of using a second procedure.

This example is based on a study comparing total laparoscopic hysterectomy (TLH) to total abdominal hysterectomy (TAH) for the treatment of early stage endometrial cancer in two modelled cohorts of 1,000 patients each in Australia ([Graves et al., 2013](#_ENREF_38)). We assume for simplicity that i) these two constitute the only alternatives and ii) resources for operations were at full capacity. Thus, the value of the bed-days used for the laparoscopic procedures are estimated in terms of the forgone abdominal procedures. The study used a higher monetary value of AU$64,000/QALY for Australia, and we used the results published for 6-month post-surgery; see Table A6. No information on the revenue was provided, which is why we omitted the corresponding approaches 3b, 3c, and 9.

| Table A6. Input data to illustrate the value of the opportunity costs: TLH in terms of TAH. | | |
| --- | --- | --- |
| Occupancy rate | 1.0^a^ |  |
| Cost-effectiveness threshold ($ per QALY) | 64,000^b^ |  |
|  |  |  |
| Patient(s) | P_1_ (TLH, n=1,000) | P_2_ (TAH) |
| Units (bed-days per patient) | 3.76 (total for 1,000 patients=3,760) | 7.31 |
| Expenditure ($ per patient) | 12,124 | 15,870 |
| Revenue ($ per patient) | - | -^c^ |
| Benefit (QALY gain per patient) | 0.86 | 0.82 |
| QALY: quality-adjusted life year, TAH: total abdominal hysterectomy, TLH: total laparoscopic hysterectomy.  All values were reflecting the situation as of 6-month post-surgery (i.e., including the LOS of readmissions, expenditure on all health services used, and the EQ-5D scores at month 6).  a: assumes full capacity and that freed beds are efficiently redeployed ([Drummond et al., 2005](#_ENREF_24)).  b: Estimated for Australia.  c: Not provided. | | |

For the 1,000 laparoscopic procedures, 3,760 bed-days were used in total at direct expenditures of AU$12.1 million (approach 7). The forgone abdominal procedures would have resulted in net monetary benefits of AU$18.8 million (approach New1); about 1.55 times the value of the direct expenditure incurred.

| Table A7. Overview of approaches to value the opportunity costs of bed-days for TLH surgery | | | |
| --- | --- | --- | --- |
| Approach | Description | Equation | Results for patient *i* |
| *Methodology A: Units of the second-best alternative forgone* | | | |
| 1 | Patient-equivalents (of TAH patients *j*) forgone | ${LOS}_{i}*\frac{1}{{LOS}_{j}} \left( *OCR \right)$ | 514.4 |
| 2 | Treatment-equivalents forgone for TAH patients *j* | $C_{i}*\frac{1}{C_{j}}$ | 764.0 |
| *Methodology B: Net benefit of the second-best alternative forgone* | | | |
|  | *Valuation in terms of money* |  |  |
| 3a | Expenditure forgone on TAH patient-equivalents | ${LOS}_{i}*\frac{C_{j}}{{LOS}_{j}}$ | $8,162,955 |
| 3b | Revenue forgone from the TAH patient-equivalents | ${LOS}_{i}*\frac{R_{j}}{{LOS}_{j}} \left( *OCR \right)$ | n/a |
| 3c | Net revenue forgone from the TAH patient-equivalents | ${LOS}_{i}*\frac{\left( R_{j}-C_{j} \right)}{{LOS}_{j}}$ | n/a |
| 5 | Gross monetary benefit forgone for the TAH patient-equivalents | ${LOS}_{i}*\frac{\left( B_{j}*\lambda\right)}{{LOS}_{j}}$ | $26,993,817 |
| New1 | Net monetary benefit forgone for the TAH patient-equivalents | ${LOS}_{i}*\frac{\left( B_{j}*\lambda-C_{j} \right)}{{LOS}_{j}}$ | $18,830,862 |
| New2 | Net monetary benefit forgone for the TAH treatment-equivalents | $C_{i}*\frac{\left( B_{j}*\lambda-C_{j} \right)}{C_{j}}$ | $27,968,471 |
|  | *Valuation in terms of health benefit (typically QALYs)* |  |  |
| 4 | Gross health benefit forgone for TAH patient-equivalents | ${LOS}_{i}*\frac{B_{j}}{{LOS}_{j}}$ | 421.8 |
| 6 | Health benefit forgone for expected second-best use | $C_{i}*\frac{1}{\lambda}$ | 189.4 |
| New3 | Net health benefit forgone for the TAH patient-equivalents | ${LOS}_{i}*\frac{\left( B_{j}-\left( \frac{C_{j}}{\lambda} \right) \right)}{{LOS}_{j}}$ | 294.2 |
| New4 | Net health benefit forgone for the TAH treatment-equivalents | $C_{i}*\frac{\left( B_{j}-\left( \frac{C_{j}}{\lambda} \right) \right)}{C_{j}}$ | 437.0 |
| *Methodology C: Expenditure of the alternative chosen* | | | |
| 7 | Expenditure for the resource consumption incurred | ${LOS}_{i}*\frac{C_{i}}{{LOS}_{i}}$ | $12,124,000 |
| 8 | Separating variable expenditure and non-monetary resource consumption | ${{LOS}_{i}*\frac{{VC}_{i}}{{LOS}_{i}} \& LOS}_{i}$ | $1,818,600 & 80 |
| *Methodology D: Expenditure of the alternative chosen + highest net benefit forgone* | | | |
| 9 | Expenditure incurred + highest net revenue forgone | ${LOS}_{i}*\left( \frac{C_{i}}{{LOS}_{i}}+\frac{\left( R_{j}-C_{j} \right)}{{LOS}_{j}} \right)$ | n/a |
| New5 | Expenditure incurred + highest net monetary benefit forgone | ${LOS}_{i}*\left( \frac{C_{i}}{{LOS}_{i}}+\frac{\left( B_{j}*\lambda-C_{j} \right)}{{LOS}_{j}} \right)$ | $30,954,862 |
| The last column illustrates the marginal opportunity costs of patients *i* consuming 3,760 bed-days.  B: (health) benefit gained per patient, C_i_: total expenditure incurred for *i*, C_j_: expenditure incurred per patient, λ: monetary value assigned to QALYs in local cost-effectiveness thresholds, LOS_i_: total bed-day consumption of *i*, LOS_j_: length of stay per patient, n/a: not available, OCR: occupancy rate, QALY: quality-adjusted life year, R: revenue per patient, TAH: total abdominal hysterectomy, TLH: total laparoscopic hysterectomy, VC: variable cost proportion of the expenditure. | | | |

Moreover, if we look at the different net benefits achievable again (see Table A8), the net monetary benefit for the forgone TAH procedures is lower than for TLH, and for the TLH procedures the “Expenditure + highest NMB forgone” is lower than the “Benefit (GMB, $ in total)” (while higher for the TAH procedure). The TAH procedures are thus a sub-optimal alternative compared to TLH, leaving TLH as the optimal choice here and the value of the opportunity costs for the 3,760 bed-days being equivalent to the second-best net monetary benefit forgone of AU$18.8 million (approach New1).

| Table A8. Opportunity cost results of the 3,760 bed-days used for TLH procedures using Approach New1. | | |
| --- | --- | --- |
| Occupancy rate | 1.0^a^ |  |
| Cost-effectiveness threshold ($ per QALY) | 64,000^b^ |  |
|  |  |  |
| Patient(s) | P_1_ (TLH, n=1,000) | P_2_ (forgone TAH procedures, n=514.4) |
| Expenditure ($ in total) | **12,124,000** | **8,162,955** |
| Benefit (GMB, $ in total) | 55,040,000 | **26,993,817** |
| NMB (benefit-expenditure, $ in total) | 42,916,000 | **18,830,862** |
| Expenditure + highest NMB forgone | **30,954,862** | 51,078,955 |
| GMB: gross monetary benefit, NMB: net monetary benefit, QALY: quality-adjusted life year, TAH: total abdominal hysterectomy, TLH: total laparoscopic hysterectomy. Figures in **bold** correspond to approaches covered in our overview table.  The situation is reversed here; the NMB is higher for P_1_ than for P_2_, and the “Expenditure + highest NMB forgone” is smaller than the “Benefit (GMB, $ in total)” for P_1_ while it is higher for P_2_. P_2_ is thus the sub-optimal alternative, and the value of the opportunity costs for the 3,760 bed-days is the forgone “Net monetary benefit ($ in total)” of $18,830,862. | | |

**References**

Abramovitch, A., Newman, W., Padaliya, B., Gill, C., & Charles, P. D. (2005). The cost of medical education in an ambulatory neurology clinic. *Journal of the National Medical Association, 97*(9), 1288–1290.

Adam, T., Evans, D. B., & Murray, C. J. (2003). Econometric estimation of country-specific hospital costs. *Cost Effectiveness and Resource Allocation, 1*(1), 3.

Aitken, R. J. (2012). Lost opportunity cost of surgical training in the Australian private sector. *ANZ Journal of Surgery, 82*(3), 145–150.

Ang, W. W., Sabharwal, S., Johannsson, H., Bhattacharya, R., & Gupte, C. M. (2016). The cost of trauma operating theatre inefficiency. *Annals of Medicine and Surgery 7*, 24–29.

Banks, M. D., Graves, N., Bauer, J. D., & Ash, S. (2010). The costs arising from pressure ulcers attributable to malnutrition. *Clinical Nutrition, 29*(2), 180–186.

Banks, M. D., Graves, N., Bauer, J. D., & Ash, S. (2013). Cost effectiveness of nutrition support in the prevention of pressure ulcer in hospitals. *European Journal of Clinical Nutrition, 67*(1), 42–46.

Basu, S., Phillips, R. S., Bitton, A., Song, Z., & Landon, B. E. (2015). Medicare Chronic Care Management Payments and Financial Returns to Primary Care Practices: A Modeling Study. *Annals of Internal Medicine, 163*(8), 580–588.

Baugh, C. W., & Bohan, J. S. (2008). Estimating observation unit profitability with options modeling. *Academic Emergency Medicine, 15*(5), 445–452.

Bayley, M. D., Schwartz, J. S., Shofer, F. S., Weiner, M., Sites, F. D., Traber, K. B., & Hollander, J. E. (2005). The financial burden of emergency department congestion and hospital crowding for chest pain patients awaiting admission. *Annals of Emergency Medicine, 45*(2), 110–117.

Binney, Z. O., Quest, T. E., Feingold, P. L., Buchman, T., & Majesko, A. A. (2014). Feasibility and economic impact of dedicated hospice inpatient units for terminally ill ICU patients. *Critical Care Medicine, 42*(5), 1074–1080.

Bodanapally, U. K., Shanmuganathan, K., Nutakki, K., Mirvis, S. E., Sliker, C. W., & Shet, N. (2013). Implementation of 24/7 radiology services in an academic medical centre level 1 trauma centre: impact on trauma resuscitation unit length of stay and economic benefit analysis. *Injury, 44*(1), 75–79.

Buist, M. D., Jaffray, L., Bell, E., Hanna, L., Weinstein, P., Kumar, S., & Grimmer, K. (2014). Utilisation of beds on the general medical unit by 'non-acute medical' patients: a retrospective study of incidence and cost in two Tasmanian regional medical hospital units. *Internal Medicine Journal, 44*(2), 171–177.

Chatterjee, A., Chen, L., Goldenberg, E. A., Bae, H. T., & Finlayson, S. R. (2010). Opportunity cost in the evaluation of surgical innovations: a case study of laparoscopic versus open colectomy. *Surgical Endoscopy, 24*(5), 1075–1079.

Chatterjee, A., McCarthy, J. E., Montagne, S. A., Leong, K., & Kerrigan, C. L. (2011). A cost, profit, and efficiency analysis of performing carpal tunnel surgery in the operating room versus the clinic setting in the United States. *Annals of Plastic Surgery, 66*(3), 245–248.

Chatterjee, A., Payette, M. J., Demas, C. P., & Finlayson, S. R. (2009). Opportunity cost: a systematic application to surgery. *Surgery, 146*(1), 18–22.

Chinai, N., Bintcliffe, F., Armstrong, E. M., Teape, J., Jones, B. M., & Hosie, K. B. (2013). Does every patient need to be discussed at a multidisciplinary team meeting? *Clinical Radiology, 68*(8), 780–784.

Connell, W. R., Samyue, T., Gibson, P. R., Lachal, S., Moore, G. T., Macrae, F. A., & Van Langenberg, D. R. (2015). Changing face of care for patients with moderate to severe inflammatory bowel disease: the role of specialist nurses in the governance of anti-TNF prescribing. *Internal Medicine Journal, 45*(11), 1161–1166.

Cookson, R., Drummond, M., & Weatherly, H. (2009). Explicit incorporation of equity considerations into economic evaluation of public health interventions. *Health Economics, Policy and Law, 4*(2), 231–245.

Coughlan, T., & O'Neill, D. (2001). General hospital resources consumed by an elderly population awaiting long-term care. *Irish Medical Journal, 94*(7), 206–208.

Coyle, D., Cheung, M. C., & Evans, G. A. (2014). Opportunity cost of funding drugs for rare diseases: the cost-effectiveness of eculizumab in paroxysmal nocturnal hemoglobinuria. *Medical Decision Making, 34*(8), 1016–1029.

Danial, J., Ballard-Smith, S., Horsburgh, C., Crombie, C., Ovens, A., Templeton, K. E., . . . Johannessen, I. (2016). Lessons learned from a prolonged and costly norovirus outbreak at a Scottish medicine of the elderly hospital: case study. *The Journal of Hospital Infection, 93*(2), 127–134.

Department of Health. (2016). NHS reference costs 2015 to 2016. Retrieved 04/02/2017, from https://[www.gov.uk/government/publications/nhs-reference-costs-2015-to-2016](http://www.gov.uk/government/publications/nhs-reference-costs-2015-to-2016)

Desaigues, B. (2001). Is Expressed WTP Consistent with Welfare Economics? A Response from 73 Cognitive Interviews. *Schweizerische Zeitschrift fur Volkswirtschaft und Statistik/Swiss Journal of Economics and Statistics, 137*(1), 35–47.

Drummond, M. F., Sculpher, M. J., Torrance, G. W., O'Brien, B. J., & Stoddart, G. L. (2005). *Methods for the economic evaluation of health care programme. Third edition*. Oxford: Oxford University Press.

Eddy, D. M. (1994). Clinical decision making: from theory to practice. Rationing resources while improving quality. How to get more for less. *The Journal of the American Medical Association, 272*(10), 817–824.

Ehreth, J. (1996). The implications for information system design of how health care costs are determined. *Medical Care, 34*(3 Suppl), MS69–MS82.

Falcone, J. L., & Feinn, R. S. (2016). A Sensitivity Analysis and Opportunity Cost Evaluation of the Surgical Council on Resident Education Curriculum. *Journal of Surgical Education, 73*(1), 24–30.

Falvo, T., Grove, L., Stachura, R., Vega, D., Stike, R., Schlenker, M., & Zirkin, W. (2007). The opportunity loss of boarding admitted patients in the emergency department. *Academic Emergency Medicine, 14*(4), 332–337.

Fuertes-Guiro, F., & Girabent-Farres, M. (2016). Opportunity cost of the dermatologist's consulting time in the economic evaluation of teledermatology. *Journal of Telemedicine and Telecare, 23*(7), 657–664.

Fuertes-Guiro, F., Girabent-Farres, M., & Viteri-Velasco, E. (2016). Opportunity Cost in the Economic Evaluation of da Vinci Robotic Assisted Surgery. *European Journal of Health Economics, 17*(3), 245–256.

Furber, S. E., & Donaldson, C. (1992). The cost of cervical cancer screening provided by a women's health nurse. *Australian Journal of Public Health, 16*(3), 226–231.

Gammon, D. C., & Lizotte, M. W. (2004). Safety and cost-effectiveness of paclitaxel administered as a 1-hour infusion versus a 3-hour infusion for various malignancies. *Journal of Infusion Nursing, 27*(4), 251–253.

Gill, B., Griffin, B., & Hesketh, B. (2013). Changing expectations concerning life-extending treatment: the relevance of opportunity cost. *Social Science & Medicine, 85*, 66–73.

Graves, N. (2004). Economics and preventing hospital-acquired infection. *Emerging Infectious Diseases, 10*(4), 561–566.

Graves, N., Birrell, F. A., & Whitby, M. (2005). Modeling the economic losses from pressure ulcers among hospitalized patients in Australia. *Wound Repair and Regeneration, 13*(5), 462–467.

Graves, N., Halton, K., Doidge, S., Clements, A., Lairson, D., & Whitby, M. (2008). Who bears the cost of healthcare-acquired surgical site infection? *The Journal of Hospital Infection, 69*(3), 274–282.

Graves, N., Harbarth, S., Beyersmann, J., Barnett, A., Halton, K., & Cooper, B. (2010). Estimating the cost of health care-associated infections: mind your p's and q's. *Clinical Infectious Diseases, 50*(7), 1017–1021.

Graves, N., Janda, M., Merollini, K., Gebski, V., Obermair, A., & Lace trial committee. (2013). The cost-effectiveness of total laparoscopic hysterectomy compared to total abdominal hysterectomy for the treatment of early stage endometrial cancer. *BMJ Open, 3*(4), e001884.

Graves, N., Nicholls, T. M., & Morris, A. J. (2003). Modeling the costs of hospital-acquired infections in New Zealand. *Infection Control & Hospital Epidemiology, 24*(3), 214–223.

Graves, N., Weinhold, D., Tong, E., Birrell, F., Doidge, S., Ramritu, P., . . . Whitby, M. (2007). Effect of healthcare-acquired infection on length of hospital stay and cost. *Infection Control & Hospital Epidemiology, 28*(3), 280–292.

Greenspoon, J. N., O'Reilly, D., Wright, J. R., Whitton, A., Sussman, J., & Birch, S. (2012). Technology resource planning in radiation oncology: application of a needs-based analytic framework to radiosurgery planning in Ontario. *Journal of Oncology Practice, 8*(6), 358–362.

Gruen, R. L., Chang, S., & MacLellan, D. G. (1996). Optimizing the hospital management of leg ulcers. *The Australian and New Zealand Journal of Surgery, 66*(3), 171–174.

Halton, K. A., Cook, D., Paterson, D. L., Safdar, N., & Graves, N. (2010). Cost-effectiveness of a central venous catheter care bundle. *PLoS One, 5*(9), e12815.

Harper, D. R. (1979). Disease cost in a surgical ward. *British Medical Journal, 1*(6164), 647–649.

Health and Social Care Information Centre. (2016). Hospital Admitted Patient Care Activity, 2015-16. Retrieved 04/02/2017, from <http://www.content.digital.nhs.uk/catalogue/PUB22378/hosp-epis-stat-admi-summ-rep-2015-16-rep.pdf>

Herzer, K. R., Niessen, L., Constenla, D. O., Ward, W. J., Jr., & Pronovost, P. J. (2014). Cost-effectiveness of a quality improvement programme to reduce central line-associated bloodstream infections in intensive care units in the USA. *BMJ Open, 4*(9), e006065.

Hjalte, K., Norinder, A., Persson, U., & Maraste, P. (2003). Health-health analysis-an alternative method for economic appraisal of health policy and safety regulation. Some empirical Swedish estimates. *Accident; Analysis and Prevention, 35*(1), 37–46.

Holdefer, R. N., & Skinner, S. A. (2016). Commentary : The value of intraoperative neurophysiological monitoring: evidence, equipoise and outcomes. *Journal of Clinical Monitoring and Computing, 31*(4), 657–664.

Hollinghurst, S., Kessler, D., Peters, T. J., & Gunnell, D. (2005). Opportunity cost of antidepressant prescribing in England: analysis of routine data. *British Medical Journal, 330*(7498), 999–1000.

Hsiao, W. C., & Stason, W. B. (1979). Toward developing a relative value scale for medical and surgical services. *Health Care Financing Review, 1*(2), 23–38.

Hsu, S. H. (2011). Cost information and pricing: empirical evidence. *Contemporary Accounting Research/Recherche Comptable Contemporaine, 28*(2), 554–579.

Hubner, C., Hubner, N. O., Hopert, K., Maletzki, S., & Flessa, S. (2014). Analysis of MRSA-attributed costs of hospitalized patients in Germany. *European Journal of Clinical Microbiology & Infectious Diseases, 33*(10), 1817–1822.

Hubner, C., Hubner, N. O., Muhr, M., Claus, F., Leesch, H., Kramer, A., & Flessa, S. (2015). Cost analysis of hospitalized Clostridium difficile-associated diarrhea (CDAD). *GMS Hygiene and Infection Control, 10*, Doc13.

Huynh, T. N., Kleerup, E. C., Raj, P. P., & Wenger, N. S. (2014). The opportunity cost of futile treatment in the ICU*. *Crit Care Med, 42*(9), 1977-1982.

Israelsson, L. A., & Wimo, A. (2000). Cost minimisation analysis of change in closure technique of midline incisions. *The European Journal of Surgery, 166*(8), 642–646.

Jenks, P. J., Laurent, M., McQuarry, S., & Watkins, R. (2014). Clinical and economic burden of surgical site infection (SSI) and predicted financial consequences of elimination of SSI from an English hospital. *The Journal of Hospital Infection, 86*(1), 24–33.

Kilgore, M. L., Steindel, S. J., & Smith, J. A. (1999). Cost analysis for decision support: the case of comparing centralized versus distributed methods for blood gas testing. *Journal of Healthcare Management, 44*(3), 207–215.

Kristensen, S. R., Siciliani, L., & Sutton, M. (2016). Optimal Price-Setting in Pay for Performance Schemes in Health Care. *Journal of Economic Behavior and Organization, 123*(0), 57–77.

Lave, J. R., Pashos, C. L., Anderson, G. F., Brailer, D., Bubolz, T., Conrad, D., . . . Provenzano, G. (1994). Costing medical care: using Medicare administrative data. *Medical Care, 32*(7 Suppl), JS77–JS89.

Lee, B. Y., Wettstein, Z. S., McGlone, S. M., Bailey, R. R., Umscheid, C. A., Smith, K. J., & Muder, R. R. (2011). Economic value of norovirus outbreak control measures in healthcare settings. *Clinical Microbiology and Infection, 17*(4), 640–646.

Leroux, E. J., Morton, J. M., & Rivas, H. (2014). Increasing access to specialty surgical care: application of a new resource allocation model to bariatric surgery. *Annals of Surgery, 260*(2), 274–278.

Lewis, J. D., & Asch, D. A. (1999). Barriers to office-based screening sigmoidoscopy: does reimbursement cover costs? *Annals of Internal Medicine, 130*(6), 525–530.

Lopman, B. A., Reacher, M. H., Vipond, I. B., Hill, D., Perry, C., Halladay, T., . . . Sarangi, J. (2004). Epidemiology and cost of nosocomial gastroenteritis, Avon, England, 2002-2003. *Emerging Infectious Diseases, 10*(10), 1827–1834.

Lucas, R., Farley, H., Twanmoh, J., Urumov, A., Evans, B., & Olsen, N. (2009). Measuring the opportunity loss of time spent boarding admitted patients in the emergency department: a multihospital analysis. *Journal of Healthcare Management, 54*(2), 117–125.

Luce, B. R., & Elixhauser, A. (1990). Estimating costs in the economic evaluation of medical technologies. *Int J Technol Assess Health Care, 6*(1), 57–75.

Lucioni, C., Mazzi, S., & Rossi, C. (2005). Proton pump inhibitors in acute treatment of reflux oesophagitis : a cost-effectiveness analysis. *Clinical Drug Investigation, 25*(5), 325–336.

Mahajan, P., Thomas, R., Rosenberg, D. R., Leleszi, J. P., Leleszi, E., Mathur, A., . . . Knazik, S. R. (2007). Evaluation of a child guidance model for visits for mental disorders to an inner-city pediatric emergency department. *Pediatric Emergency Care, 23*(4), 212–217.

Mangan, J. L., Walsh, C., Kernohan, W. G., Murphy, J. S., Mollan, R. A., McMillen, R., & Beverland, D. E. (1992). Total joint replacement: implication of cancelled operations for hospital costs and waiting list management. *Quality in Health Care, 1*(1), 34–37.

Mayhew, M., Ickx, P., Newbrander, W., Stanekzai, H., & Alawi, S. A. (2015). Long and short Integrated Management of Childhood Illness (IMCI) training courses in Afghanistan: a cross-sectional cohort comparison of post-course knowledge and performance. *Int J Health Policy Manag, 4*(3), 143–152.

McIntosh, E., Donaldson, C., & Ryan, M. (1999). Recent advances in the methods of cost-benefit analysis in healthcare. Matching the art to the science. *PharmacoEconomics, 15*(4), 357–367.

McKay, D. R. (2013). Costs of regional and general anesthesia: what the plastic surgeon needs to know. *Clinics in Plastic Surgery, 40*(4), 529–535.

Mentzakis, E., Ryan, M., & McNamee, P. (2011). Using discrete choice experiments to value informal care tasks: exploring preference heterogeneity. *Health Economics, 20*(8), 930–944.

Mutters, N. T., Gunther, F., Frank, U., & Mischnik, A. (2016). Costs and possible benefits of a two-tier infection control management strategy consisting of active screening for multidrug-resistant organisms and tailored control measures. *The Journal of Hospital Infection, 93*(2), 191–196.

National Institute for Health and Care Excellence. (2013). *Guide to the methods of technology appraisal 2013*. London: NICE.

Navarro, A. M., & Kaplan, R. M. (1996). Mammography screening: prospects and opportunity costs. *Womens Health, 2*(4), 209–233.

Nguyen, K. H., Chaboyer, W., & Whitty, J. A. (2015). Pressure injury in Australian public hospitals: a cost-of-illness study. *Australian Health Review, 39*(3), 329–336.

Nicks, B. A., & Manthey, D. M. (2012). The impact of psychiatric patient boarding in emergency departments. *Emergency Medicine International, 2012*, 360308.

Norovirus Working Party. (2012). Guidelines for the management of norovirus outbreaks in acute and community health and social care settings. https://[www.gov.uk/government/publications/norovirus-managing-outbreaks-in-acute-and-community-health-and-social-care-settings](http://www.gov.uk/government/publications/norovirus-managing-outbreaks-in-acute-and-community-health-and-social-care-settings)

O'Brien, K., & Donato, R. (1993). Hospital acquired rota virus infection: the economics of prevention. *Australian Health Review, 16*(3), 245–267.

Oliver, A. (2002). Accounting for the missing opportunity costs in incremental cost-outcome analysis. *Applied Health Economics and Health Policy, 1*(4), 191–196.

Otter, J. A., Burgess, P., Davies, F., Mookerjee, S., Singleton, J., Gilchrist, M., . . . Holmes, A. H. (2016). Counting the cost of an outbreak of carbapenemase-producing Enterobacteriaceae: an economic evaluation from a hospital perspective. *Clin Microbiol Infect, 23*(3), 188–196.

Pashayan, N., Lyratzopoulos, G., & Mathur, R. (2006). Cost-effectiveness of primary offer of IVF vs. primary offer of IUI followed by IVF (for IUI failures) in couples with unexplained or mild male factor subfertility. *BMC Health Services Research, 6*, 80.

Peeraully, R., Henderson, K., & Davies, B. (2016). Emergency readmissions to paediatric surgery and urology: The impact of inappropriate coding. *Annals of the Royal College of Surgeons of England, 98*(4), 250–253.

Perroca, M. G., Jerico Mde, C., & Facundin, S. D. (2007). Surgery cancelling at a teaching hospital: implications for cost management. *Revista Latino-Americana de Enfermagem, 15*(5), 1018–1024.

Piednoir, E., Borderan, G. C., Borgey, F., Thibon, P., Lesellier, P., Leservoisier, R., . . . Le Coutour, X. (2010). Direct costs associated with a hospital-acquired outbreak of rotaviral gastroenteritis infection in a long term care institution. *The Journal of Hospital Infection, 75*(4), 295–298.

Richardson, J., Iezzi, A., Sinha, K., Khan, M. A., & McKie, J. (2014). An instrument for measuring the social willingness to pay for health state improvement. *Health Economics, 23*(7), 792–805.

Rodney, W. M., Hardison, D., Rodney-Arnold, K., & McKenzie, L. (2006). Impact of deliveries on the office practice of family medicine. *Journal of the National Medical Association, 98*(10), 1685–1690.

Rojo-Manaute, J. M., Capa-Grasa, A., Del Cerro-Gutierrez, M., Martinez, M. V., Chana-Rodriguez, F., & Martin, J. V. (2012). Sonographically guided intrasheath percutaneous release of the first annular pulley for trigger digits, part 2: randomized comparative study of the economic impact of 3 surgical models. *Journal of Ultrasound in Medicine, 31*(3), 427–438.

Rosemurgy, A. S., Ryan, C. E., Klein, R. L., Wood, T. W., Co, F., & Ross, S. B. (2016). Financial Benefits of a Hepatopancreaticobiliary Program. *The American Surgeon, 82*(5), 380–385.

Russell, L. B. (1992). Opportunity costs in modern medicine. *Health Affairs (Millwood), 11*(2), 162–169.

Saka, O., Serra, V., Samyshkin, Y., McGuire, A., & Wolfe, C. C. (2009). Cost-effectiveness of stroke unit care followed by early supported discharge. *Stroke, 40*(1), 24–29.

Salomon, J. A., Haagsma, J. A., Davis, A., de Noordhout, C. M., Polinder, S., Havelaar, A. H., . . . Vos, T. (2015). Disability weights for the Global Burden of Disease 2013 study. *The Lancet Global Health, 3*(11), e712–e723.

Santillan, A., Govan, L., Zahurak, M. L., Diaz-Montes, T. P., Giuntoli, R. L., 2nd, & Bristow, R. E. (2008). Feasibility and economic impact of a clinical pathway for pap test utilization in Gynecologic Oncology practice. *Gynecologic Oncology, 109*(3), 388–393.

Sasor, S. E., Flores, R. L., Wooden, W. A., & Tholpady, S. (2013). The cost of intraoperative plastic surgery education. *Journal of Surgical Education, 70*(5), 655–659.

Southey, D., Pullinger, D., Loggos, S., Kumari, N., Lengyel, E., Morgan, I., . . . Luckraz, H. (2015). Discharge of thoracic patients on portable digital suction: Is it cost-effective? *Asian Cardiovascular & Thoracic Annals, 23*(7), 832–838.

Spencer, I. C., Coast, J., Spry, P. G., Smith, L., & Sparrow, J. M. (1995). The cost of monitoring glaucoma patients by community optometrists. *Ophthalmic and Physiological Optics, 15*(5), 383–386.

Stewardson, A. J., Harbarth, S., Graves, N., & Timber Study Group. (2014). Valuation of hospital bed-days released by infection control programs: a comparison of methods. *Infection Control and Hospital Epidemiology, 35*(10), 1294–1297.

Taheri, P. A., Wahl, W. L., Butz, D. A., Iteld, L. H., Michaels, A. J., Griffes, L. C., & Greenfield, L. (1998). Trauma service cost: the real story. *Annals of Surgery, 227*(5), 720–725.

Turner-Stokes, L. (2007). Cost-efficiency of longer-stay rehabilitation programmes: can they provide value for money? *Brain Injury, 21*(10), 1015–1021.

Turner-Stokes, L., Bill, A., & Dredge, R. (2012). A cost analysis of specialist inpatient neurorehabilitation services in the UK. *Clinical Rehabilitation, 26*(3), 256–263.

Turner-Stokes, L., Poppleton, R., Williams, H., Schoewenaars, K., & Badwan, D. (2012). Using the UKROC dataset to make the case for resources to improve cost-efficiency in neurological rehabilitation. *Disability and Rehabilitation, 34*(22), 1900–1906.

Turner-Stokes, L., Sutch, S., & Dredge, R. (2012). Healthcare tariffs for specialist inpatient neurorehabilitation services: rationale and development of a UK casemix and costing methodology. *Clinical Rehabilitation, 26*(3), 264–279.

van de Vooren, K., Curto, A., & Garattini, L. (2014). Curing cervical cancer or preventing it: A case of opportunity cost in the long run? *Vaccine, 32*(51), 6867–6869.

Wagner, T., Fydrich, T., Stiglmayr, C., Marschall, P., Salize, H. J., Renneberg, B., . . . Roepke, S. (2014). Societal cost-of-illness in patients with borderline personality disorder one year before, during and after dialectical behavior therapy in routine outpatient care. *Behaviour Research and Therapy, 61*, 12–22.

Ward, M. J., Eckman, M. H., Schauer, D. P., Raja, A. S., & Collins, S. (2011). Cost-effectiveness of telemetry for hospitalized patients with low-risk chest pain. *Academic Emergency Medicine, 18*(3), 279–286.

Wariyapola, C., Littlehales, E., Abayasekara, K., Fall, D., Parker, V., & Hatton, G. (2016). Improving the quality of vascular surgical discharge planning in a hub centre. *Annals of the Royal College of Surgeons of England, 98*(4), 275–279.

Young, N., Kinsella, S., Raio, C. C., Nelson, M., Chiricolo, G., Johnson, A., . . . Sama, A. (2010). Economic impact of additional radiographic studies after registered diagnostic medical sonographer (RDMS)-certified emergency physician-performed identification of cholecystitis by ultrasound. *Journal of Emergency Medicine, 38*(5), 645–651.
